# Transplantation of oral mucosal epithelial cells seeded on decellularized and lyophilized amniotic membrane for the regeneration of injured endometrium

**DOI:** 10.1186/s13287-019-1179-z

**Published:** 2019-03-21

**Authors:** Xing Chen, Jingtao Sun, Xiaoyu Li, Lele Mao, Lei Cui, Wenpei Bai

**Affiliations:** 1 Department of Obstetrics and Gynecology, Beijing Shijitan Hospital, Capital Medical University, Beijing, 100038 China; 2 Department of Plastic Surgery, Beijing Shijitan Hospital, Capital Medical University, Beijing, 100038 China

**Keywords:** Intrauterine adhesion, Oral mucosal epithelial cells, Decellularized and lyophilized amniotic membrane, Fibrosis

## Abstract

**Background:**

Intrauterine adhesion (IUA) is characterized by progressive intrauterine fibrosis as a consequence of trauma to the basal layer of the endometrium. In an attempt to relieve IUA, many approaches have been applied in the clinic but show limited effects. In this study, we investigated the effect of autologous oral mucosal epithelial cells (OMECs) seeded on decellularized and lyophilized amniotic membrane (DL-AM) on preventing the development of IUA in a rat model.

**Methods:**

IUA model was established by surgical scraping of the endometrium in the left uteri (the right uteri were kept as control) of SD rats. Wounds were randomly treated as unrepaired (IUA group), repaired with DL-AM alone (DL-AM group), and DL-AM seeded with autologous OMECs (DL-AM+OMECs group), respectively, in a total of 54 rats (*n* = 18 each). Uterus samples were harvested for histological and immunohistochemical evaluation after 3, 7, 14, and 28 days (*n* = 3 in each time point) of operations.

**Results:**

After surgery, the uterine cavity was observed to be filled with extensive fibrosis in the IUA and DL-AM groups, respectively, while a lower ratio of the fibrotic area was identified in the DL-AM transplantation group. Transplantation of OMECs seeded on DL-AM significantly reduced fibrosis of IUA with recovered uterine cavity and regenerated epithelium and endometrial glands as determined by CK-18 immunostaining. OMECs transplantation resulted in extensive cellular proliferation as revealed by the Ki-67 immunofluorescent staining exhibited. Meanwhile, microvessel density was significantly increased in uteri that received OMECs transplantation, which was concomitant with elevated expression of vascular endothelial growth factor. The pregnancy test (*n* = 6 in each group) showed successful conception in the OMEC-transplanted uteri, but not in the IUA and DL-AM groups.

**Conclusions:**

Engineered epithelium developed from DL-AM seeded with OMECs showed great potential in preventing progression of intrauterine adhesion by improved endometrial epithelium regeneration.

**Electronic supplementary material:**

The online version of this article (10.1186/s13287-019-1179-z) contains supplementary material, which is available to authorized users.

## Background

Intrauterine adhesion (IUA), also known as the Asherman syndrome, is characterized by progressive intrauterine fibrosis as a consequence of trauma to the basal layer of the endometrium. Not only does IUA affect women’s menstruation (amenorrhea, hypomenorrhea, and dysmenorrhea), but also damages their fertility including secondary infertility and recurrent miscarriages, which has become a wide concern worldwide [1–3]. The prevalence of IUA is significantly increased with the explosive growth of hysteroscopic operations and artificial abortions in recent years. In an attempt to relieve IUA, many approaches such as hysteroscopic adhesiolysis, intrauterine contraceptive device, Foley catheter balloon, and hyaluronic acid injection have been applied in the clinic [4]. However, these approaches showed limited effects in ameliorating endometrial fibrosis, in particular for patients with moderate-to-severe IUA that has a high recurrence rate.

In recent years, tissue-engineering technology has developed rapidly and become a promising alternative in repairing tissue defect and even in regenerating solid organs. As one of the most popular biological materials, amniotic membrane (AM) has been widely utilized to support cell proliferation and differentiation, thus facilitating tissue regeneration [5]. It has been shown that the placement of AM after surgery reduced IUA as evidenced by decreased American Fertility Society (AFS) IUA scoring (decreased from 10 to 2) and improved pictorial blood loss assessment chart score (increased from 11.5 ± 5.4 to 25.1 ± 5.9), respectively [6]. Removal of the cellular component in AM (decellularized AM (dAM)) has been shown to eliminate immunological rejections and promote better cell adhesion, proliferation, and colonization of corneal epithelial cells and thus make it an ideal matrix in tissue engineering and regenerative medicine [7–9].

Oral mucosal epithelial cells (OMECs) are sufficiently available, can be harvested by minimally invasive procurement, and possess high proliferation potential in vitro, all of which make OMECs an ideal seed cell for tissue engineering [10]. By transplantation of OMEC sheets immediately after endometrial damage, Kuramoto et al. observed the presence of uterine cavities without fibrosis formation for 8 days [11]. However, without a supportive scaffold, it is difficult to manipulate the fragile OMEC sheet in surgery and adhesion of cell sheet to the hollow uterus cavity is limited. Furthermore, long-term preventive effects of OMECs on IUA need to be observed. Previous studies have shown that OMECs spread and proliferated well on decellularized AM [8, 12]. Decellularized AM that seeded with OMECs has been transplanted to restore damaged cornea with rapid re-epithelialization [8, 12]. However, whether seeding OMECs on decellularized AM have preventive effects on IUA remains unknown.

In the present study, we seeded autologous OMECs on the decellularized and lyophilized amniotic membrane (DL-AM) to generate engineered epithelia. The preventive efficacy of OMECs seeded on DL-AM was evaluated in IUA rat models for as long as 4 weeks.

## Materials and methods

### Ethics

All surgical procedures were performed on female Sprague-Dawley rats of 8 weeks (Beijing Vital River Laboratory Animal Technology, China). Rats were housed under conditions in a natural light-dark cycle (12-h light and 12-h dark) with free access to food and water. All animals were treated according to the guidelines of the Institutional Animal Care and Use Committee of Beijing Shijitan Hospital, which were complied with the ARRIVE (Animal Research: Reporting of In Vivo Experiments) guidelines. All animal procedures were approved by the Laboratory Animal Ethics Committee of Beijing Shijitan Hospital. All researchers involved in animal experiments possessed animal experimentation licenses issued by the Beijing Association on Laboratory Animal Care (BALAC). Isolation of human AM from the placenta was obtained from pregnant who underwent cesarean sections and approved by the Ethics Committee of Beijing Shijitan Hospital with prior written informed consent.

### Preparation of decellularized AM

Fresh human AM was obtained under sterile conditions from patients who are seronegative for syphilis, human immunodeficiency virus, and hepatitis B and C virus after caesarian sections performed in Beijing Shijitan Hospital with prior written informed consent. Preparation of DL-AM was performed according to reports by Koizumi et al. [9]. Briefly, fresh AM mechanically peeled from the chorion membrane was washed extensively with 0.9% sodium chloride solution for three times. After cut into 2.5 × 2.5 cm square pieces, AM was treated with 0.02% ethylenediaminetetraacetic acid (EDTA) solution for 2 h at 37 °C, followed by gentle mechanical scraping with a cell scraper and subjected to rinsing in phosphate-buffered saline (PBS) for three times to remove cellular debris. Decellularized AM was dried in a lyophilizer (FD-1A-50, Biocool, China) for 8 h, vacuum packed and sterilized by γ-ray (Co-60) irradiation.

### Isolation and seeding of autologous OMECs on DL-AM

Oral mucosal epithelium (approximately 4 × 5 mm) were surgically harvested for isolation of autologous OMECs. Briefly, oral mucosal epithelium were digested with Dispase II (1 mg/ml, Gibco) overnight at 4 °C. Epithelial layers were peeled off with forceps and digested with 0.25% trypsin–0.02% EDTA solution (Gibco) for 3 min at 37 °C with shaking to obtain cell suspensions. After the addition of 10% fetal bovine serum (Coring) to terminate enzyme activity, the supernatant was discarded and primary cells were resuspended and cultured in serum-free oral keratinocyte medium (ScienCell). OMECs (1 × 10^6^/ml) were seeded and cultured on DL-AM at 37 °C in a 5% CO_2_:95% air incubator for 10 days and changed the medium once daily.

### Surgical transplantation of OMECs

**Fig. 1:**
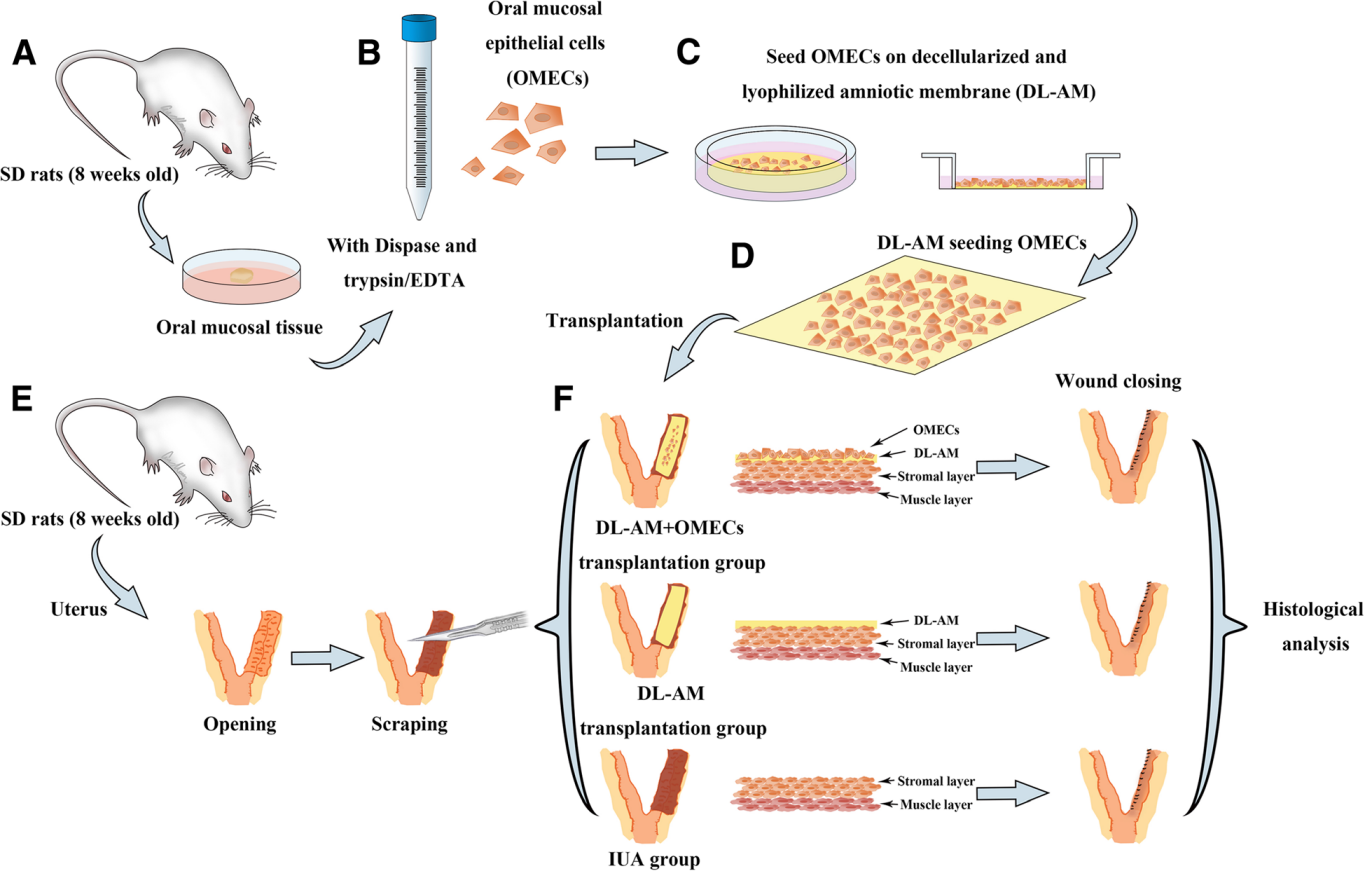
Schematic illustration of experimental procedures. (A) Oral mucosal tissue was resected. (B) The tissue was treated with Dispase and trypsin/EDTA for isolation of oral mucosal epithelial cells (OMECs). (C, D) Isolated OMECs were seeded and incubated on decellularized and lyophilized amniotic membrane (DL-AM) for 14 days and transplanted to damaged uteri. (E) The left uterus was scraped to establish an IUA rat model. (F) DL-AM seeded with OMECs and DL-AM alone were transplanted. After surgery, uteri were harvested and subjected to histological analysis

A total of 54 rats were randomly divided into three groups: IUA, DL-AM, and DL-AM+OMECs groups (*n* = 18 for each group, 12 were sacrificed at 3, 7, 14, and 28 days post-surgery for histological and immunohistochemical evaluation and the other 6 for pregnancy test 4 weeks after surgery). After the administration of 10% chloral hydrate by intraperitoneal injection, a vertical incision through the abdominal midline in the rat was made to expose the uteri. The left uteri of each animal received endometrial scraping while the contralateral right uteri were kept as control. In the IUA group, the uterus was exposed and received endometrial scraping to the depth of the muscular layer by a no. 21 razor blade in the left uteri. The uterine wound was then closed without treatment. In the DL-AM group, DL-AM was introduced to cover the damaged areas by suturing it to wound edge. In the DL-AM+OMECs group, scraped wounds were covered by the transplantation of DL-AM harboring autologous OMECs. On 3, 7, 14, and 28 days post-operation (*n* = 3 for each time point), uteri were collected for gross, histological, and immunohistochemical evaluation (Fig. 1).

### Histology, immunohistochemistry, and immunofluorescence

Samples were fixed in 4% paraformaldehyde, embedded in paraffin, and cut into 5-μm slices for H&E staining. Picro-sirius red staining was performed according to the manufacturer’s instruction to evaluate the degree of fibrosis by Image-Pro Plus v6.0. Under polarized light microscopy, fibers of collagen types I and III show color of red and green respectively. Microvessel density (MVD) was analyzed according to the method by Meng et al. [13] by immunohistochemical staining of CD34, which was an indicator of endothelial cells of microvessels.

For immunohistochemical staining, sections were fixed in 3% hydrogen peroxide solution for 15 min to block endogenous peroxidase reactivity and incubated with primary antibodies (anti-vascular endothelial growth factor (VEGF), ab46154, Abcam; anti-CD34, bs-0646R, Bioss) overnight at 4 °C. Horseradish peroxidase (HRP)-labeled secondary antibody was incubated, followed by addition of 3-3′-diaminobenzidine tetrahydrochloride solution to visualize the reaction products. For immunofluorescence, primary antibodies (anti-Ki-67, ab15580, Abcam; anti-cytokeratin 18(CK-18), ab668, Abcam) were incubated after blocking for 1 h by donkey serum. Either fluorescein isothiocyanate or tetramethyl rhodamine-labeled secondary antibody was incubated for immunofluorescence staining, and 4,6-diamidino-2-phenylindole was used to stain the nucleus. Sections were observed using confocal laser scanning microscopy (A1, Nikon, Japan). OMECs were labeled by fluorescent dye DIO before seeding on DL-AM.

Numbers of endometrial glands and microvessel density that characterized by immunohistochemical staining of CD34 were calculated in high-power field (HPF) (× 400) under a light microscope (BX51, Olympus) [14]. Four fields were selected in each section, and two sections were selected for counting for each rat.

### Scanning electron microscopy

The specimen was fixed in 1% glutaraldehyde for 24 h and washed three times in PBS (15 min per time). After treatment with 1% osmium tetroxide for 2 h, the specimen was dehydrated by sequential alcohol treatment and by increasing ethanol-n-pentyl acetate series up to 100% before samples were subjected to drying. Specimens were coated with gold before visualized on scanning electron microscopy (S-3400N, Hitachi, Japan).

### Statistical analysis

The data were presented as mean ± standard deviation and analyzed by SPSS 22.0. Statistical analysis was performed by Student’s *t* test for comparisons of different groups. Values of *P* < 0.05 were considered as statistical significance.

## Results

### AM decellularization and OMEC seeding

**Fig. 2:**
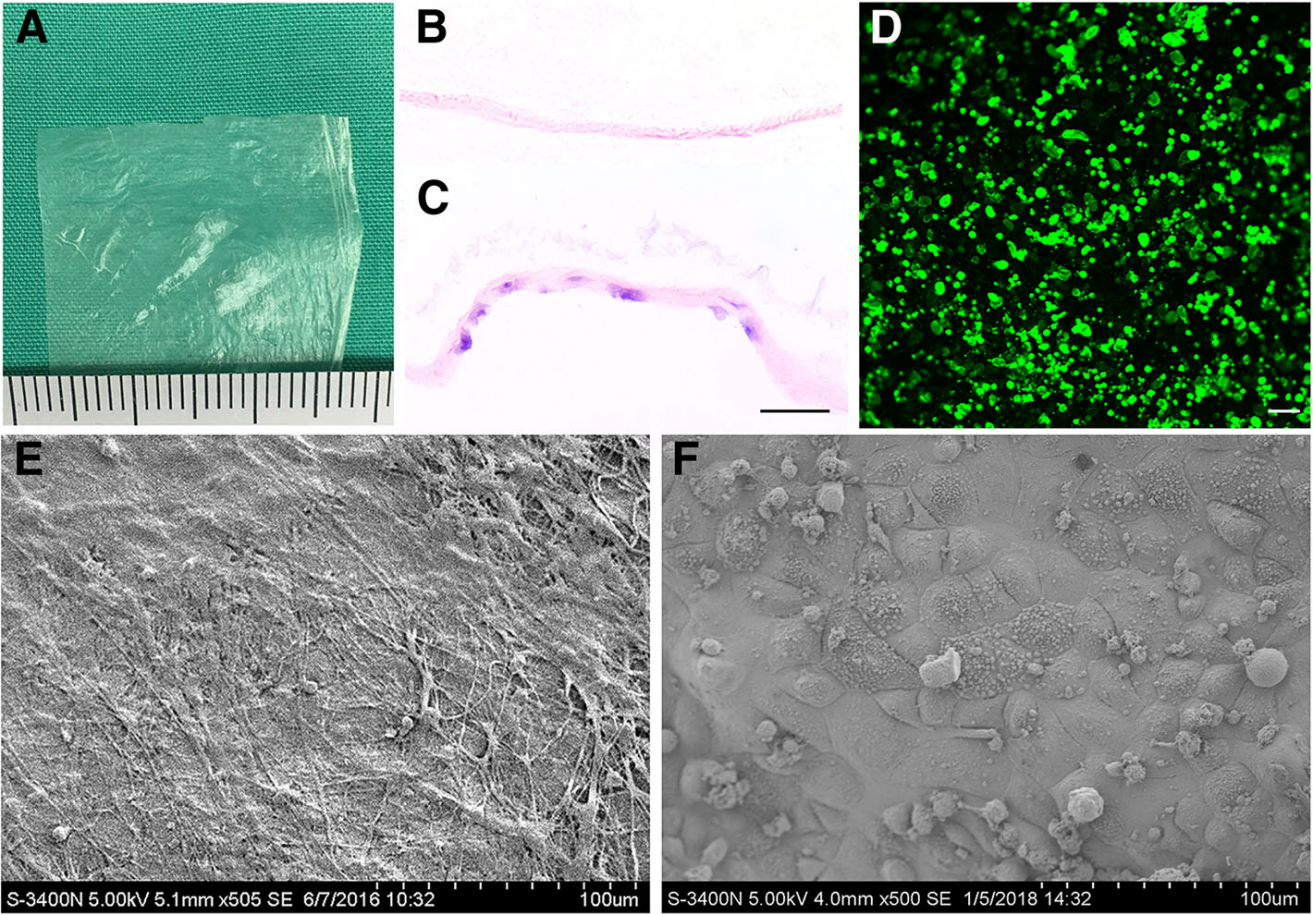
Seeding of oral mucosal epithelial cells (OMECs) on decellularized amniotic membrane (AM). **a**–**c** Gross view and H&E staining of DL-AM and DL-AM seeding OMECs. **d** OMEC labeling using DIO. **e**, **f** DL-AM and DL-AM seeding OMECs by scanning electron microscopy. Bar = 50 μm

H&E staining showed the absence of epithelial cellular structure on DL-AM, while a continuous layer of epithelial cells was observed after seeding OMECs on DL-AM for 10 days. By scanning electron microscopy, only collagen fibers were observed in DL-AM while the structure of epithelial cells was found in DL-AM that seeded with OMECs. By confocal laser scanning microscopy, OMECs that pre-labeled with fluorescent dye DIO was observed to form a uniform layer of epithelium covering DL-AM which was green (Fig. 2).

### Histology and fibrosis evaluation

**Fig. 3:**
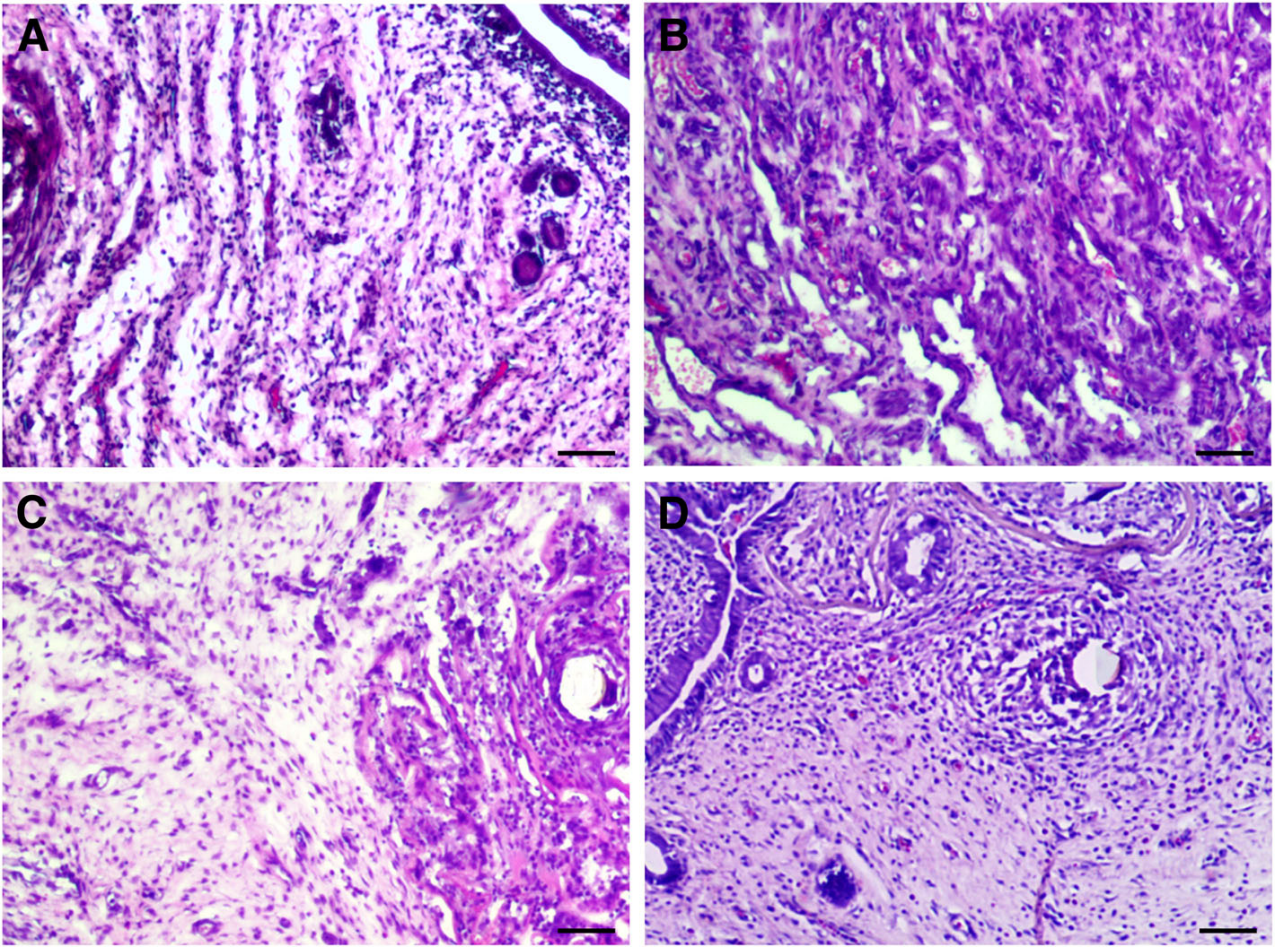
H&E staining of uteri at day 28 post-surgery among groups. (**a**) H&E staining of the uterus of control group. Uterine cavity was filled with fibrous tissue in the IUA (**b**) and DL-AM groups (**c**). In the DL-AM +OMECs group, regeneration of endometrium was found after 28 days of surgery (**d**). Bar = 100 μm

As shown in Fig. 3, at day 28 post-surgery, the absence of uterine cavity and endometrial epithelium was observed in the IUA and DL-AM groups by H&E staining, which was instead occupied with collagen fibers. In the DL-AM+OMECs group, the regeneration of endometrium and appearance of the uterine cavity was detected after 28 days of surgery (results on 3, 7, and 14 days were shown in Additional file 1: Figure S1; Additional file 2: Figure S2; and Additional file 3: Figure S3).

**Fig. 4:**
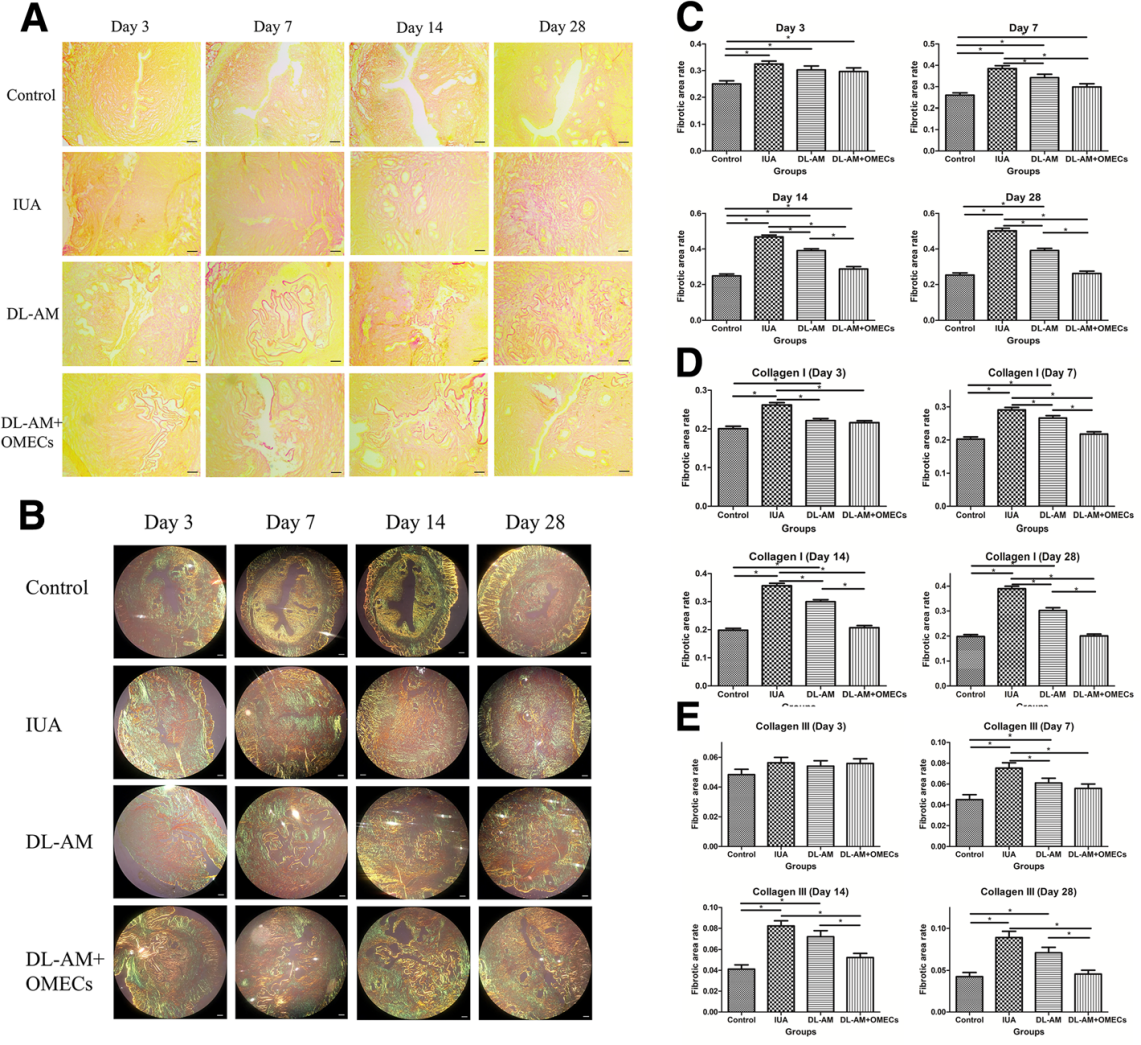
Fibrosis of IUA was reduced with the transplantation of OMECs. **a** Picro-sirus red staining and **b** polarized light microscopy observation after 3, 7, 14, and 28 days of surgeries (× 40). **c** The percentage of fibrotic areas of uteri after 3, 7, 14, and 28 days of operations. *P < 0.05. **d** The proportion of the areas of collagen type I and **e** type III among groups. *P < 0.05

Under light microscopy, collagen fibers were stained red by picro-sirius red staining, while endometrial glands and muscular layers were stained yellow. At 3, 7, 14, and 28 days after surgery, recovery of the uterine cavity was not observed which was occupied with dense fibers that stained red in the IUA group (Fig. 4a). We next observed sections of picro-sirius red staining under polarized light microscopy, by which collagen type I showed orange-red or yellow, while collagen type III demonstrated green (Fig. 4b).

The ratio of the fibrotic area which was defined as the ratio of endometrial fibrotic area to the whole endometrial area was counted. At 3 days after operations, the ratio of the fibrotic area in IUA, DL-AM, and DL-AM+OMECs groups were significantly higher than that in the control group (*P* < 0.05). At 7 days after surgery, the ratio of the fibrotic area in the IUA group was higher compared to the other groups (*P* < 0.05). After 14 days of transplantation, the ratio of the fibrotic area in the DL-AM+OMECs group ((28.65 ± 1.35)%) was smaller than that in the IUA ((46.78 ± 0.98)%) and DL-AM groups ((39.03 ± 1.07)%) (*P* < 0.05), but it was still higher than that in the control group ((24.82 ± 1.06)%) (*P* < 0.05). After modeling for 28 days, the ratio of fibrotic area in the DL-AM+OMECs group ((26.18 ± 1.28)%) was significantly decreased compared to the IUA ((50.19 ± 1.45)%) and DL-AM groups ((39.08 ± 1.21)%) (*P* < 0.05) and there was no significant difference compared to the control group ((25.29 ± 1.18)%) (*P* > 0.05) (Fig. 4c).

Under polarized light microscopy, the proportion of the area of collagen type I area in the DL-AM+OMECs group was significantly decreased as compared with that in the IUA and DL-AM groups, respectively, (*P* < 0.05) at 7, 14, and 28 days after surgery (Fig. 4d). There was no significant difference in the proportion of collagen type III area detected among groups after 3 days of operation (*P* > 0.05). However, the proportions of the areas of collagen type III in the DL-AM+OMECs group were significantly lower than those in the IUA group after 7, 14, and 28 days of surgeries and lower than those in the DL-AM group after 14 and 28 days of surgeries (*P* < 0.05) (Fig. 4e).

### Repair of injured endometrium with OMEC transplantation

**Fig. 5:**
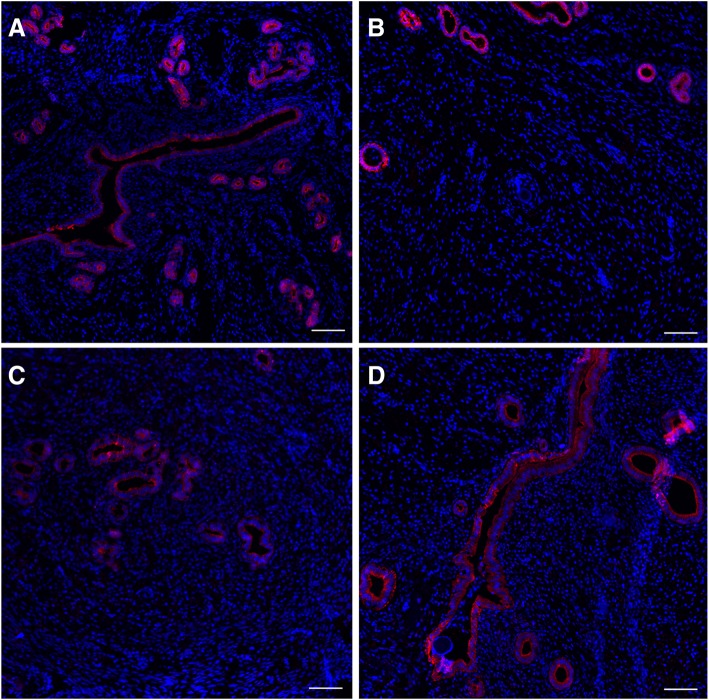
Immunofluorescent staining of CK-18 in the control group (**a**), IUA group (**b**), DL-AM group (**c**), and DL-AM+OMECs group (**d**) after 28 days of operations. Bar = 100 μm

Immunofluorescent staining showed CK-18 was specifically expressed within the epithelium of endometrial glands and endometrial cells in the normal uterus. In the DL-AM+OMECs group, CK-18-positive cells were observed in both neogenerated endometrial cells and epithelium of endometrial glands as that in normal uterine, while expression of CK-18 was merely observed in endometrial glands in the IUA and DL-AM groups, respectively. The number of endometrial glands increased with the healing of endometrial wounds in the DL-AM+OMECs group. After 28 days of transplantation, the number of endometrial glands in the DL-AM+OMECs group was significantly higher than that in the IUA and DL-AM groups (*P* < 0.05) and showed no statistical difference with normal controls (*P* > 0.05) (Fig. 5, Additional file 4: Figure S4 for results of 3 days; Additional file 5: Figure S5 for results of 7 days, Additional file 6: Figure S6 for results of 14 days and Additional file 7: Figure S7).

**Fig. 6:**
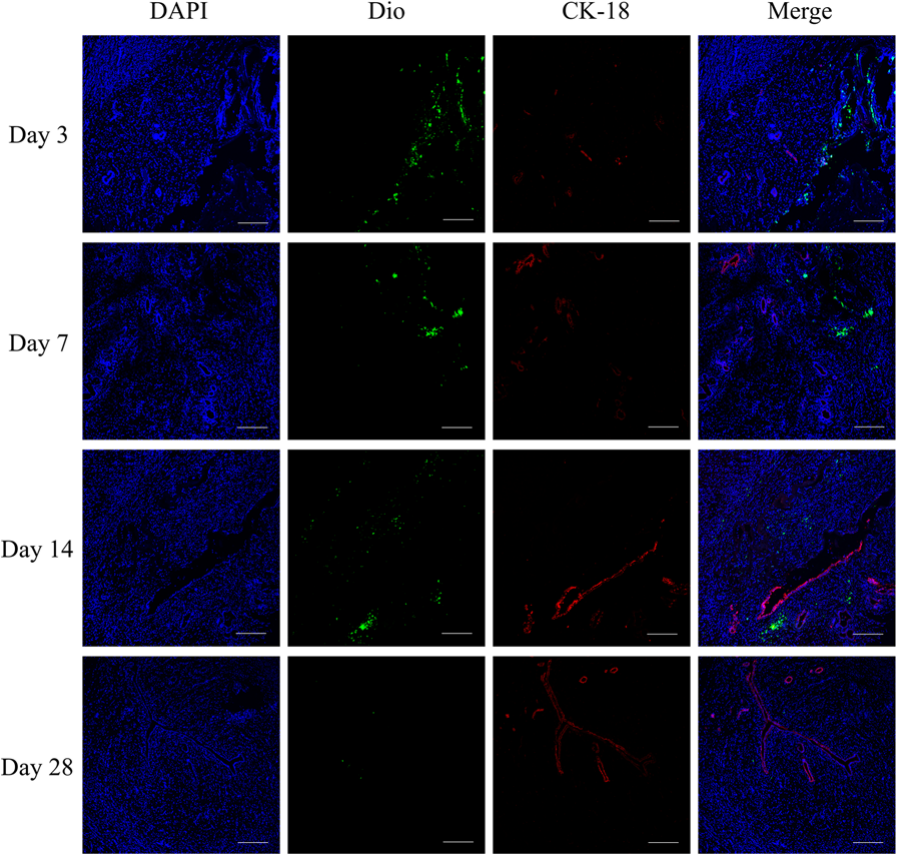
Co-staining of (red) DIO-labeled OMECs (green) after 3, 7, 14, and 28 days of operations. Bar = 100 μm

To detect whether regenerated epithelial cells were originated from transplanted cells in the DL-AM+OMECs group, we labeled OMECs with DIO fluorescent dye before transplantation and traced its expression in vivo. Survival of DIO-labeled cells was detected in generated sub-endometrial layer after 3, 7, 14, and 28 days. With immunofluorescent staining of CK-18, it was found that there was no co-expression of CK-18 in DIO-labeled cells within the duration of 28 days, indicating that regenerated endometrial cells were not of transplanted OMEC origin (Fig. 6).

**Fig. 7:**
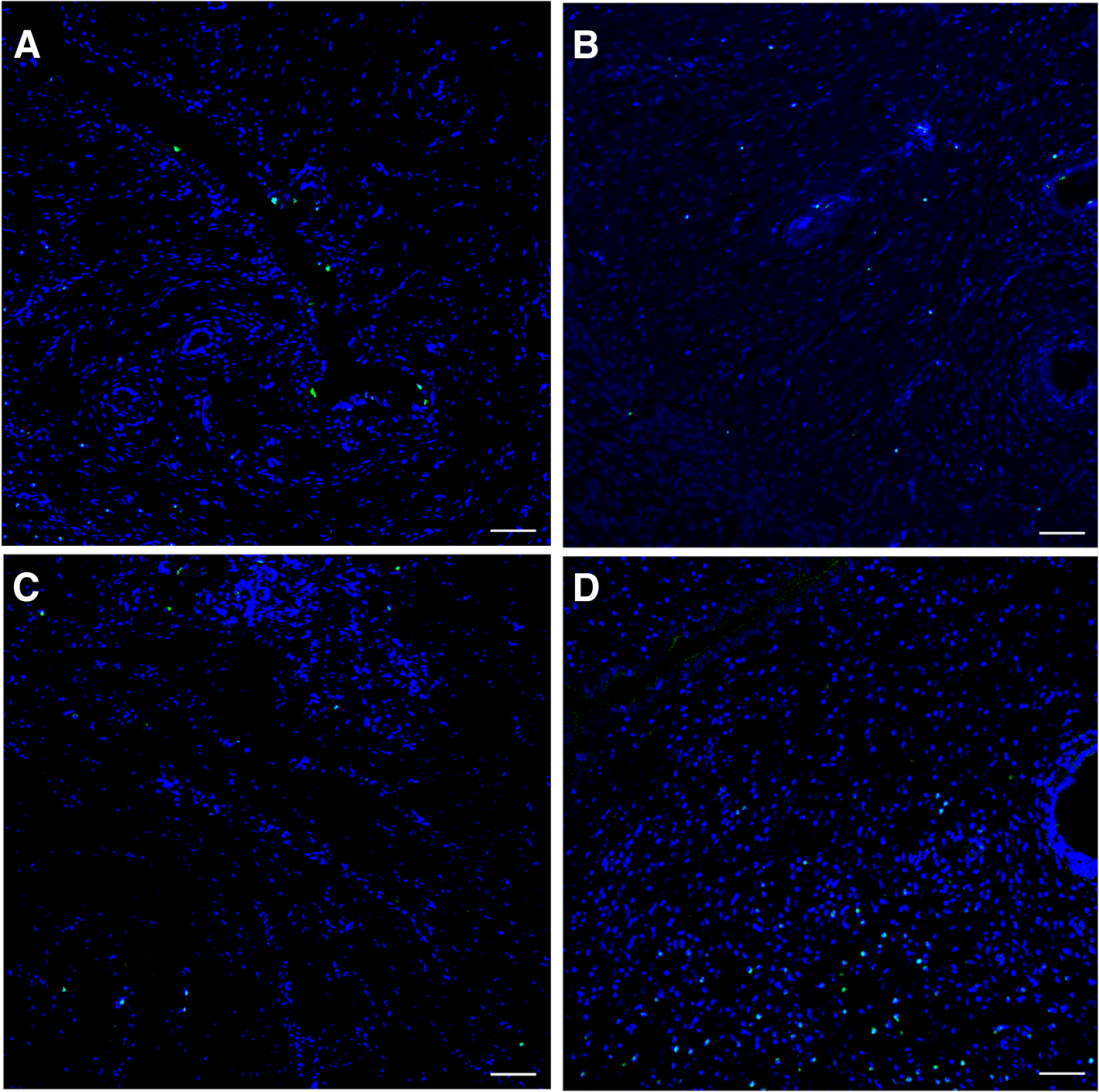
Immunofluorescent staining of Ki-67 after 28 days of operation among groups. (**a**) represented control group, (**b**) IUA group, (**c**) DL-AM group and (**d**) DL-AM+OMECs group, resepectively. Bar = 100 μm

To further explore the impact of OMEC transplantation on cellular proliferation, we calculated the percentage of Ki-67(+) cells in healing uteri. We found an evident increase of Ki-67(+) cells in OMEC-engrafted uteri which mainly localized in endometrial stromal cells and epithelium of endometrial glands and endometrium, while there was no statistical difference between the IUA and DL-AM groups (*P* > 0.05) (Fig. 7 for results of 28 days; Additional file 8: Figure S8 for results of 3 days; Additional file 9: Figure S9 for results of 7 days; Additional file 10: Figure S10 for results of 14 days; and Additional file 11: Figure S11).

**Fig. 8:**
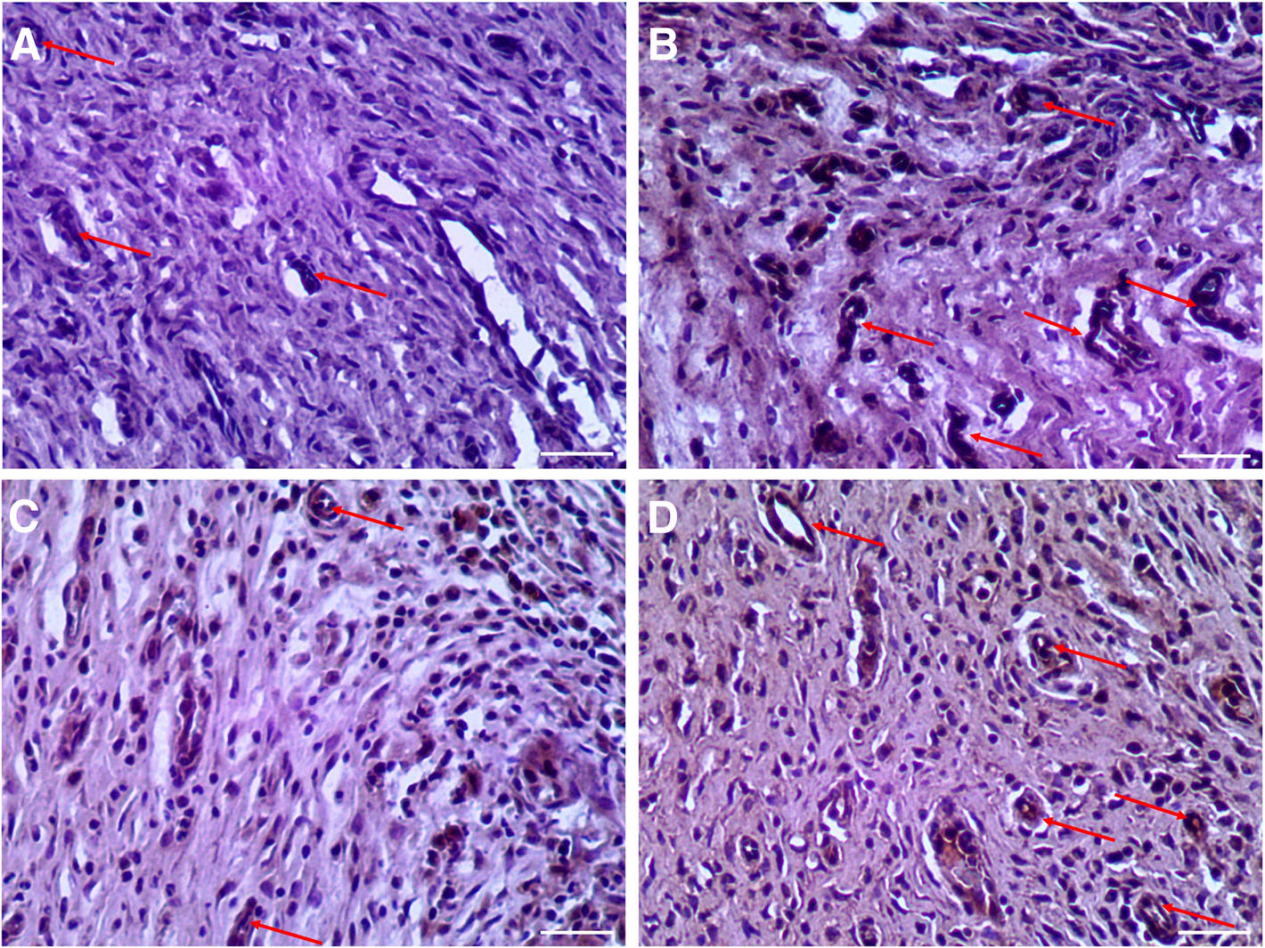
Immunohistochemical staining of CD34 at 28 days after operations among groups. (**a**) represented control group, (**b**) IUA group, (**c**)DL-AM group and (**d**) DL-AM+OMECs group, resepectively. Red arrows indicated microvessels which were positive for CD34. Bar = 100 μm

**Fig. 9:**
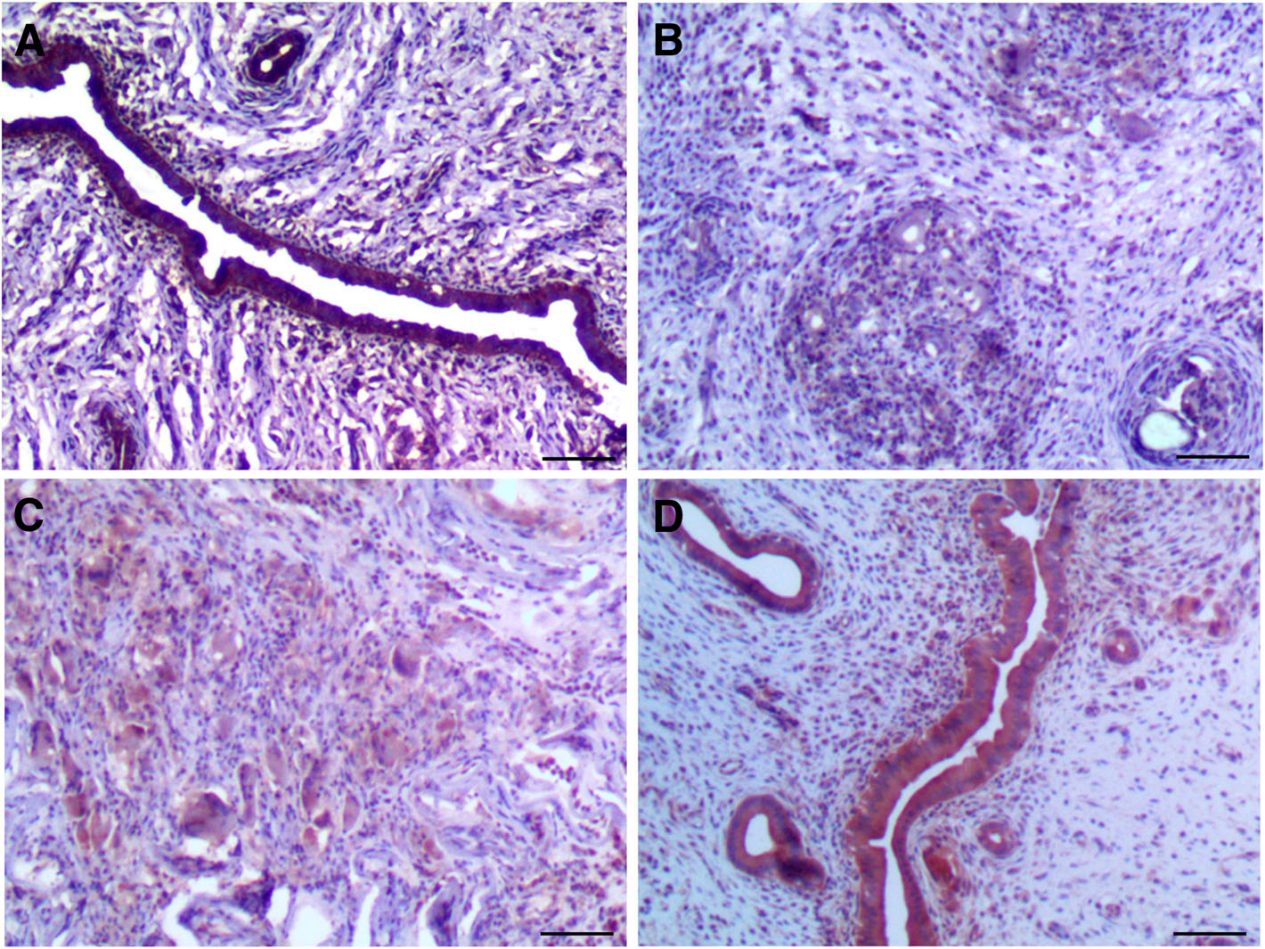
Immunohistochemical staining of VEGF among groups at 28 days after operations. (**a**) represented control group, (**b**) IUA group, (**c**) DL-AM group and (**d**) DL-AM+OMECs group, resepectively. Bar = 100 μm

To further investigate neovascularization in repaired endometrium, capillary densities were assessed by immunofluorescent staining for CD34 according to Meng et al. [15]. The MVD in the DL-AM+OMECs group was significantly higher than that in the IUA and DL-AM groups, respectively, at 7, 14, and 28 days after surgery (*P* < 0.05). Vascular density in the OMEC-treated endometrium was comparable to that in the normal uterine after 14 and 28 days (Fig. 8 for results of 28 days; Additional file 12: Figure S12 for results of 3 days; Additional file 13: Figure S13 for results of 7 days; Additional file 14: Figure S14 for results of 14 days; and Additional file 15: Figure S15). Given that VEGF plays a critical role in neoangiogenesis, expression of VEGF was determined by immunohistochemical staining in the endometrium at 3, 7, 14, and 28 days after surgery. There was a gradual increase of VEGF expression with the healing of wounds in each group, respectively. Expression of VEGF in the DL-AM+OMECs group maintained the highest level among all three groups, showing significantly higher than that in the IUA and DL-AM groups (*P* < 0.05), respectively, from days 7 to 28 after surgery. Meanwhile, at 14 and 28 days, expression of the VEGF in the DL-AM group was higher than that in the IUA group (*P* < 0.05) (Fig. 9 for results of 28 days; Additional file 16: Figure S16 for results of 3 days; Additional file 17: Figure S17 for results of 7 days; Additional file 18: Figure S18 for results of 14 days; and Additional file 19: Figure S19).

### Recovery of fertility in IUA rats that received OMEC transplantation

**Fig. 10:**
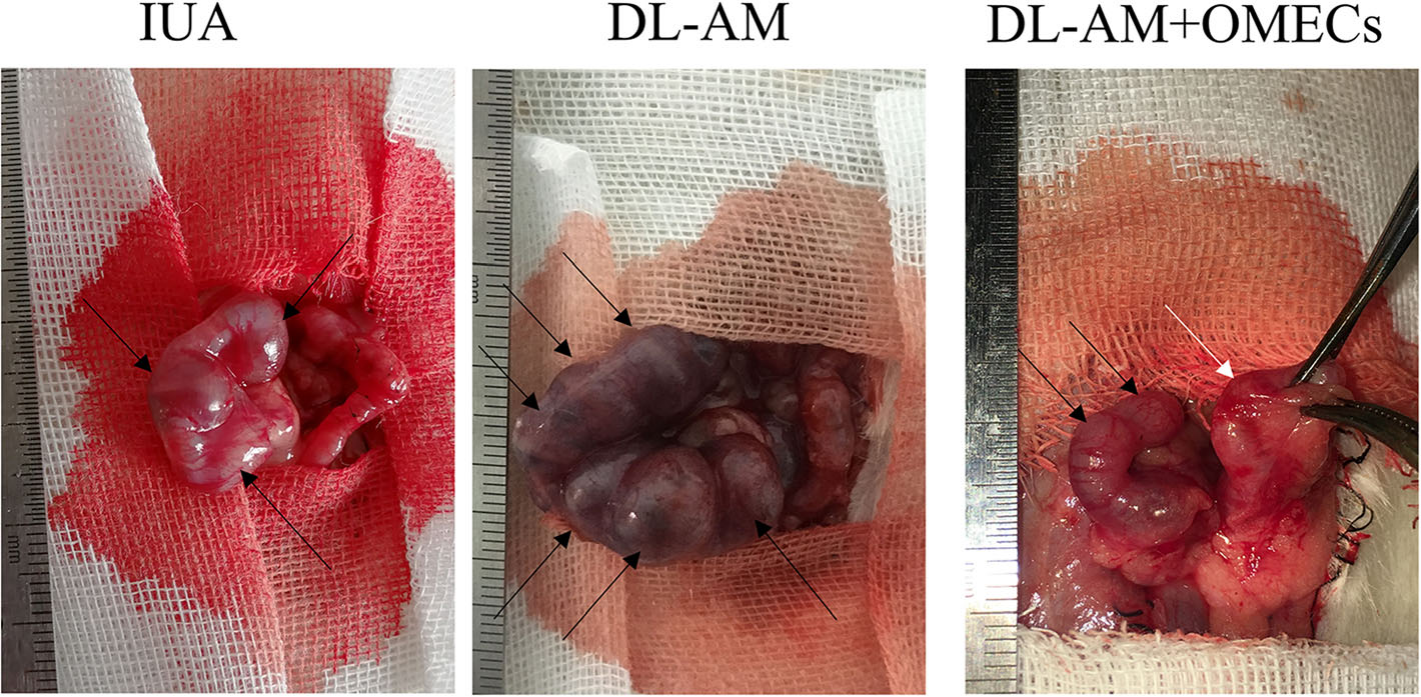
Pregnant outcomes of OMEC transplantation. Rats were bred after 28 days of OMEC transplantation. The left uteri that were conceived as normal pregnant control (black arrows). No embryo was found in the IUA and DL-AM groups (right uteri). In the DL-AM+OMECs group, the embryo could be clearly identified although there were only 1–2 embryos (white arrow) compared with the control side (6–8 embryos)

To observe the functional improvement in IUA rats after OMEC transplantation, 18 female rats (*n* = 6 in each group) were bred after 4 weeks of OMEC transplantation. In the normal control group, all uteri conceived (100%), while none of the uteri in the IUA and DL-AM groups conceived (0%). In the DL-AM+OMECs group, although 3 rats conceived (50%), the average number of embryos transplanted on uteri (1–2 embryos in each uterus) was significantly less than in the control group (6–8 embryos in each uterus) (Fig. 10).

## Discussion

With the growth of artificial abortion and other intrauterine operations, an increasing number of cases of IUA is described worldwide. The reason why IUA happens has been attributed to a failure in endometrium regeneration and instead of the development of fibrotic tissue after surgery. Therefore, an ideal therapy of IUA should achieve three goals: inhibition of adhesion formation, promotion of endometrial regeneration, and improvement in pregnancy outcome.

AM is a thin (0.02–0.05 mm) membrane located in the inner side of the placenta, containing no vessels, nerves, lymphatics, and muscles. The advantage of using AM as a biological material includes non-immunogenic, inexpensiveness, ease of availability, anti-inflammatory, anti-bacterial, and little ethical problems [15], which makes AM an ideal scaffold for delivering cells in tissue engineering. In the prevention of IUA, Amer et al. [16] showed significant improvement in adhesion grade with amnion graft compared with intrauterine balloon alone. Fresh AM maintains the whole structure and cytokines but cannot be preserved for a long time and has potential infectious risks.

In order to seed cells more efficiently on AM, the process of decellularization was performed in a variety of studies. Although the expression of growth factor was reduced after decellularization, removal of epithelial cells of AM has been reported to eliminate immunological rejection and promote better cell adhesion, proliferation, and differentiation [8–12]. Moreover, a robust proliferation of OMECs that seeded was observed after seeded on decellularized AM [8], suggesting good biocompatibility of this biomaterial to OMECs. In addition to decellularization, the procedure of lyophilization makes the amniotic membrane be readily restored and reduces the risk of pathogen transmission after Co-60 irradiation. Based on these results, we choose DL-AM as a scaffold in our study to deliver seeded OMECs in the prevention of IUA.

Kuramoto et al. [11] reported the preventive effect of OMEC sheet transplantation on IUA in an 8-day follow-up study. However, whether transplantation of OMECs has a long-term effect on the inhibition of IUA with the neogeneration of the endometrium and endometrial glands remained unknown. We thus investigated whether the combination of OMECs with DL-AM has a synergistic effect in prevention against in a long-term study.

Histologically, IUA is a condition in which the endometrial stroma is largely replaced by fibrous tissue [1]. With the progression of intrauterine fibrosis, significantly increased deposition of collagen type I was detected. Moreover, the ratio of collagen type I to type III increased in fibrotic tissue [17]. In order to show intrauterine fibrosis, we performed picro-sirius staining to detect deposition of collagen fibers. Under polarized light microscopy, collagen types I and III could be clearly clarified.

With the transplantation of DL-AM alone, the ratio of fibrotic area and the proportion of the area of collagen types I and III were observed decreased compared to IUA group, suggesting an anti-fibrotic property of DL-AM. Seeding of OMECs on DL-AM further significantly decreased the ratio of fibrotic area and the proportion of the area of collagen types I and III, showing no significant differences with normal control uterus, indicating that OMEC transplantation significantly enhanced the anti-fibrotic effect of DL-AM after the damage to the endometrium.

Although DL-AM graft reduced endometrial fibrosis, no regeneration of endometrial epithelium and epithelial glands were observed in DL-AM transplantation alone group, while we found that transplantation of DL-AM that harbored OMECs greatly enhanced endometrial epithelium regeneration with more endometrial glands distributed within sub-endometrial layers after 14 and 28 days of surgery. Meanwhile, more percentage of Ki-67-positive cells were observed in the DL-AM+OMECs group, locating mainly within the epithelium of endometrial glands, suggesting that neogenerated endometrium may arise from resident gland epithelial cells in the damaged uterus. Higher expression of Ki-67 in stromal cells suggests a potential of stromal cells trans-differentiate into endometrial epithelial cells and thus regenerate endometrium directly. However, this needs further investigation.

Transplantation of epithelial cells harvested from oral mucosa has shown great promise in a clinic in treating corneal stem cell deficiency [10], releasing esophageal stricture [18] and repairing bladder [19] and urethral epithelial defects [20]. However, the mechanism of OMECs transplantation on repairing an ectopic epithelium defect remains largely unknown. Many studies showed that cultured OMECs contain epithelial stem cells or progenitor cells characterized by expression of p63 and integrin β1 [17, 21, 22], and thus, it is suggested that stem or progenitor cells in OMECs may involve in the repair of epithelium damage in the recipient site. In studies of ocular reconstruction using OMECs, it was found that the gene expression profile of cultured OMECs remained oral but not corneal [23], while keratin expression profile of OMECs was found be altered [24, 25] after transplanted to the ocular surface, despite not fully differentiated into corneal epithelial cells [25], indicating that microenvironment of ocular surface might contribute to this change in cell phenotype. In this study, we detected endometrial epithelial-specific CK-18 expression in generated epithelium but not in DIO-labeled OMECs, indicating that the newly generated epithelium is not mainly originated from grafted OMECs. However, it cannot fully exclude the possibility that OMECs trans-differentiate into endometrial epithelial cells when transplanted into the uterus.

Paracrine factors secreted from grafted OMECs may also play a role in the regeneration of endometrium. It has shown that transplantation of amniotic mesenchymal stem cells (hAMSCs) accelerated endometrium regeneration with downregulation of pro-inflammatory cytokines, such as interleukin-1β, tumor necrosis factor-α, and interleukin-8, and upregulation of anti-inflammatory cytokines, such as interleukin-6 and interleukin-10, as well as VEGF, basic fibroblast growth factor, hepatocyte growth factor [26]. In our experiments, increased expression of VEGF was detected with OMEC transplantation, suggesting that secretion of growth factors either from OMECs or cells in microenvironment may improve tissue regeneration. However, further studies need to be performed to trace the origin of epithelial cells in newly generated endometrium and to explore mechanisms of endometrium regeneration resulted from OMEC transplantation.

VEGF is a critical inducer of angiogenesis by which re-epithelialization of the endometrium is improved. Compared with the DL-AM alone and IUA group, a higher density of microvessels was identified in uteri of the DL-AM+OMECs group, with more expression of VEGF as determined by immunohistochemistry. Previous studies showed that, in response to tissue damage, angiogenesis was promoted by engrafted mesenchymal stem cells and thus triggers growth and differentiation of local cells to regenerate the tissues in a paracrine manner [27, 28]. In this study, improved angiogenesis in OMEC-transplanted uterus is therefore a result of increased VEGF expression which facilitates repairing of damaged endometrium. Similar results were reported in cornea transplanted with OMECs, a higher density of microvessels was detected.

Functional reconstruction is the ultimate goal for IUA treatment. In the present pregnancy test, although 50% rats in the DL-AM+OMECs group was conceived, there was still less transplanted on uteri compared with the control group. Although neogenerated endometrium was observed by histology, whether it had the same characteristics of normal endometrium such as estrogen-sensitive and embryo receptivity was unknown. Endometrial receptivity for regenerated endometrium, which is critical for embryo implantation, needed to be further evaluated [29]. More rats should be involved in the pregnancy test to further confirm the functional regeneration of endometrium. Because of the remaining DL-AM in the stromal layer of endometrium found in sections after 28 days of transplantation, longer time for observation of regeneration of endometrium might also be needed. Recent advances in based on the application of novel three-dimensional scaffolds have opened up new perspectives for the field of tissue engineering [30].

## Conclusion

In conclusion, it was demonstrated that OMECs carried by DL-AM were effective in preventing fibrosis with improved regeneration of endometrium and endometrial glands in the rat model of IUA. However, the exact mechanism of OMECs on injured endometrium and whether OMEC transplantation could promote functional regeneration needed further confirmation. In humans, transplantation of OMECs carried by DL-AM offers the possibility to prevent not only IUA after hysteroscopic operations but also re-adhesion after synechiotomy for IUA.

## Additional files

Additional file 1: **Figure S1.** H&E staining of uteri at days 3 post-surgery in control group (A), IUA group (B), DL-AM group (C) and DL-AM+OMECs group (D). Regeneration of endometrium was not found. Bar = 100 μm. (TIF 5975 kb)

Additional file 2: **Figure S2.** H&E staining of uteri at days 7 post-surgery in control group (A), IUA group (B), DL-AM group (C) and DL-AM+OMECs group (D). Regeneration of endometrium was not found. Bar = 100 μm. (TIF 6333 kb)

Additional file 3: **Figure S3.** H&E staining of uteri at days 14 post-surgery in control group (A), IUA group (B), DL-AM group (C) and DL-AM+OMECs group (D). Regeneration of endometrium was found in DL-AM+OMECs group. Bar = 100 μm. (TIF 6258 kb)

Additional file 4: **Figure S4.** Immunofluorescent staining of CK-18 in control group (A), IUA group (B), DL-AM group (C) and DL-AM+OMECs group (D) after 3 days of operations. Bar = 100 μm. (TIF 2882 kb)

Additional file 5: **Figure S5.** Immunofluorescent staining of CK-18 in control group (A), IUA group (B), DL-AM group (C) and DL-AM+OMECs group (D) after 7 days of operations. Bar = 100 μm. (TIF 2969 kb)

Additional file 6: **Figure S6.** Immunofluorescent staining of CK-18 in control group (A), IUA group (B), DL-AM group (C) and DL-AM+OMECs group (D) after 14 days of operations. Bar = 100 μm. (TIF 2869 kb)

Additional file 7: **Figure S7.** The comparison of the number of endometrial glands in control group, IUA group, DL-AM group and DL-AM+OMECs group after 3, 7, 14 and 28 days of surgeries. *P<0.05. (TIF 4577 kb)

Additional file 8: **Figure S8.** Immunofluorescent staining of Ki-67 after 3 days of operation. Bar = 100 μm. (TIF 1516 kb)

Additional file 9: **Figure S9.** Immunofluorescent staining of Ki-67 after 7 days of operation. Bar = 100 μm. (TIF 1141 kb)

Additional file 10: **Figure S10.** Immunofluorescent staining of Ki-67 after 14 days of operation. Bar = 100 μm. (TIF 1126 kb)

Additional file 11: **Figure S11.** Comparison of the percentage of Ki-67(+) cells in different groups after 3, 7, 14 and 28 days of operation. *P < 0.05. (TIF 4446 kb)

Additional file 12: **Figure S12.** Immunohistochemical staining of CD34 at 3 days after operations. Red arrows indicated microvessels which were positive for CD34. Bar = 100 μm. (TIF 6665 kb)

Additional file 13: **Figure S13.** Immunohistochemical staining of CD34 at 7 days after operations. Red arrows indicated microvessels which were positive for CD34. Bar = 100 μm. (TIF 6370 kb)

Additional file 14: **Figure S14.** Immunohistochemical staining of CD34 at 14 days after operations. Red arrows indicated microvessels which were positive for CD34. Bar = 100 μm. (TIF 6599 kb)

Additional file 15: **Figure S15.** Comparison of the MVD among different groups. *P < 0.05. (TIF 4666 kb)

Additional file 16: **Figure S16.** Immunohistochemical staining of VEGF among groups at 3 days after operations. Bar = 100 μm. (TIF 5500 kb)

Additional file 17: **Figure S17.** Immunohistochemical staining of VEGF among groups at 7 days after operations. Bar = 100 μm. (TIF 5863 kb)

Additional file 18: **Figure S18.** Immunohistochemical staining of VEGF among groups at 14 days after operations. Bar = 100 μm. (TIF 5741 kb)

Additional file 19: **Figure S19.** Histogram of comparison of VEGF expression among different groups. Bar = 50 μm.*P < 0.05. (TIF 4586 kb)

## Abbreviations

AM: Amniotic membrane
CK: Cytokeratin
dAM: Decellularized amniotic membrane
DL-AM: Decellularized and lyophilized amniotic membrane
EDTA: Ethylenediaminetetraacetic acid
HPF: High-power field
IUA: Intrauterine adhesion
MVD: Microvessel density
OMECs: Oral mucosal epithelial cells
VEGF: Vascular endothelial growth factor

## References

[1] Yu D, Wong YM, Cheong Y, Xia E, Li TC (2008). Asherman syndrome-one century later. Fertil Steril.

[2] March CM (2011). Management of Asherman’s syndrome. Reprod BioMed Online.

[3] March CM (2011). Asherman’s syndrome. Semin Reprod Med.

[4] Lin X, Wei M, Li TC, Huang Q, Huang D, Zhou F, Zhang S (2013). A comparison of intrauteine balloon, intrauterine contraceptive device and hyaluronic acid gel in the prevention of adhesion reformation following hysteroscopic surgery for Asherman syndrome. Eur J Obste Gynecol Reprod Biol.

[5] Riau AK, Beuerman RW, Lim LS, Mehta JS (2010). Preservation, sterilization and de-epithelialization of human amniotic membrane for use in ocular surface reconstruction. Biomaterials.

[6] Gan L, Duan H, Sun FQ, Xu Q, Tang YQ, Wang S (2017). Efficacy of freeze-dried amnion graft following hysteroscopic adhesiolysis of severe intrauterine adhesions. Int J Gynaecol Obstet.

[7] Qi F, Yoshida T, Koike T, Aizawa H, Shimane T, Li Y, Yamada S, Okabe M, Nikaido T, Kurita H (2016). Construction and characterization of human oral mucosa equivalent using hyper-dry amniotic membrane as a matrix. Arch Oral Biol.

[8] Koizumi N, Fullwood NJ, Bairaktaris G, Inatomi T, Kinoshita S, Quantock AJ (2000). Cultivation of corneal epithelial cells on intact and denuded human amniotic membrane. Invest Ophthalmol Vis Sci.

[9] Koizumi N, Rigby H, Fullwood NJ, Kawasaki S, Tanioka H, Koizumi K, Kociok N, Joussen AM, Kinoshita S (2007). Comparison of intact and denuded amniotic membrane as a substrate for cell-suspension culture of human limbal epithelial cells. Graefes Arch Clin Exp Ophthalmol.

[10] Nishida K, Yamato M, Hayashida Y, Watanabe K, Yamamoto K, Adachi E, Na- gai S, Kikuchi A, Maeda N, Watanabe H, Okano T, Tano Y (2004). Corneal reconstruction with tissue-engineered cell sheets composed of autologous oral mucosal epithelium. N Engl J Med.

[11] Kuramoto G, Takagi S, Ishitani K, Shimizu T, Okano T, Matsui H (2015). Preventive effect of oral mucosal epithelial cell sheets on intrauterine adhesions. Hum Reprod.

[12] Prabhasawat P, Ekpo P, Uiprasertkul M, Chotikavanich S, Tesavibul N, Porn panich K, Luemsamran P (2016). Long-term result of autologous cultivated oral mucosal epithelial transplantation for severe ocular surface disease. Cell Tissue Bank.

[13] Meng H, Song Y, Zhu J, Liu Q, Lu P, Ye N, Zhang Z, Pang Y, Qi J, Wu H (2016). LRG1 promotes angiogenesis through upregulating the TGFβ1 pathway in ischemic rat brain. Mol Med Rep.

[14] Li J, Du S, Sheng X, Liu J, Cen B, Huang F, He Y (2016). MicroRNA-29b inhibits endometrial fibrosis by regulating the Sp1-TGF-β1/Smad-CTGF axis in a rat model. Reprod Sci.

[15] Toda A, Okabe M, Yoshida T, Nikaido T (2007). The potential of amniotic membrane/amnion-derived cells for regeneration of various tissues. J Pharmacol Sci.

[16] Amer MI, Abd-El-Maeboud KH, Abdelfatah I, Salama FA, Abdallah AS (2010). Human amnion as a temporary biologic barrier after hysteroscopic lysis of severe intrauterine adhesions: pilot study. J Minim Invasive Gynecol.

[17] Huang Y, de Boer WB, Adams LA, MacQuillan G, Rossi E, Rigby P, Raftopoulos SC, Bulsara M, Jeffrey GP (2013). Image analysis of liver collagen using sirius red is more accurate and correlates better with serum fibrosis markers than trichrome. Liver Int.

[18] Ohki T, Yamato M, Ota M, Takagi R, Murakami D, Kondo M, Sasaki R, Namiki H, Okano T, Yamamoto M (2012). Prevention of esophageal stricture after endoscopic submucosal dissection using tissue-engineered cell sheets. Gastroenterology.

[19] Watanabe E, Yamato M, Shiroyanagi Y, Tanabe K, Okano T (2011). Bladder augmentation using tissue-engineered autologous oral mucosal epithelial cell sheets grafted on demucosalized gastric flaps. Transplantation.

[20] Li C, Xu YM, Liu ZS, Li HB (2013). Urethral reconstruction with tissue engineering and RNA interference techniques in rabbits. Urology.

[21] Sugiyama H, Yamato M, Nishida K, Okano T (2014). Evidence of the survival of ectopically transplanted oral mucosal epithelial stem cells after repeated wounding of cornea. Mol Ther.

[22] Soma T, Hayashi R, Sugiyama H, Tsujikawa M, Kanayama S, Oie Y, Nishida K (2014). Maintenance and distribution of epithelial stem/progenitor cells after corneal reconstruction using oral mucosal epithelial cell sheets. PLoS One.

[23] Hayashida Y, Nishida K, Yamato M, Watanabe K, Maeda N, Watanabe H, Kikuchi A, Okano T, Tano Y (2005). Ocular surface reconstruction using autologous rabbit oral mucosal epithelial sheets fabricated ex vivo on a temperature-responsive culture surface. Invest Ophthalmol Vis Sci.

[24] Kolli S, Ahmad S, Mudhar HS, Meeny A, Lako M, Figueiredo FC (2014). Successful application of ex vivo expanded human autologous oral mucosal epithelium for the treatment of total bilateral limbal stem cell deficiency. Stem Cells.

[25] Bardag-Gorce F, Oliva J, Wood A, Hoft R, Pan D, Thropay J, Makalinao A, French SW, Niihara Y (2015). Carrier-free cultured autologous oral mucosa epithelial cell sheet (CAOMECS) for corneal epithelium reconstruction: a histological study. Ocul Surf.

[26] Gan L, Duan H, Xu Q, Tang Y, Li J, Sun F, Wang S (2017). Human amniotic mesenchymal stromal cell transplantation improves endometrial regeneration in rodent models of intrauterine adhesions. Cytotherapy.

[27] Zhang L, Li Y, Guan C, Tian S, Lv X, Li J, Ma X, Xia H (2018). Therapeutic effect of human umbilical cord-derived mesenchymal stem cells on injured rat endometrium during its chronic phase. Stem Cell Res Ther.

[28] Meirelles Lda S, Fontes AM, Covas DT, Caplan AI (2009). Mechanisms involved in the therapeutic properties of mesenchymal stem cells. Cytokine Growth Factor Rev.

[29] Bellver J, Simon C (2018). Implantation failure of endometrial origin: what is new?. Curr Opin Obstet Gynecol.

[30] Santamaria X, Mas A, Cervelló I, Taylor H, Simon C (2018). Uterine stem cells: from basic research to advanced cell therapies. Hum Reprod Update.

