# Supplementary figures and images for: Transplantation of oral mucosal epithelial cells seeded on decellularized and lyophilized amniotic membrane for the regeneration of injured endometrium
Source: Stem Cell Res Ther. 2019 Mar 21;10:107. doi: 10.1186/s13287-019-1179-z (PMC6429789; doi:10.1186/s13287-019-1179-z)

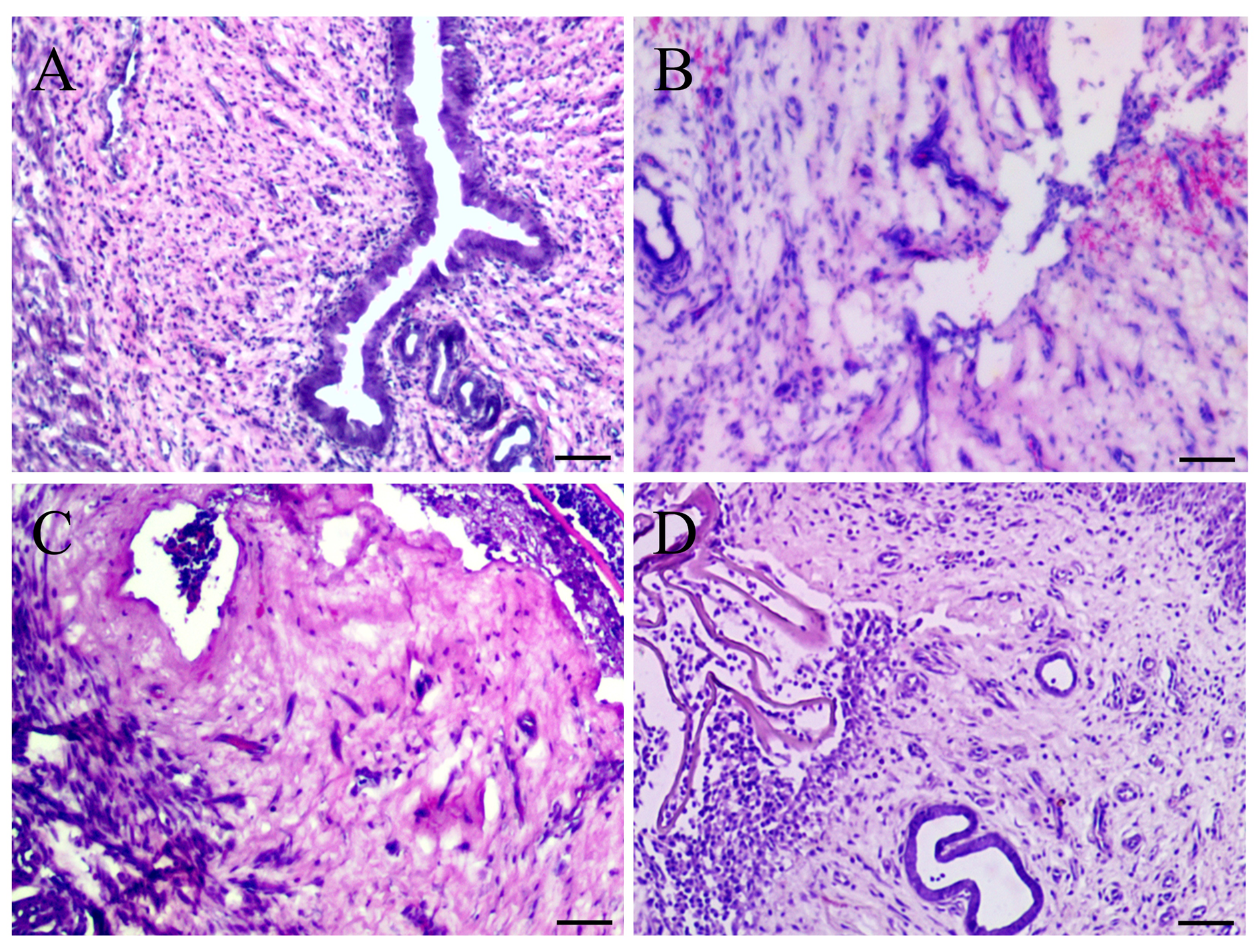

Supplement: Supplementary file 1 — Figure S1. H&E staining of uteri at days 3 post-surgery in control group (A), IUA group (B), DL-AM group (C) and DL-AM+OMECs group (D). Regeneration of endometrium was not found. Bar = 100 μm. (TIF 5975 kb) [file 13287_2019_1179_MOESM1_ESM.tif]

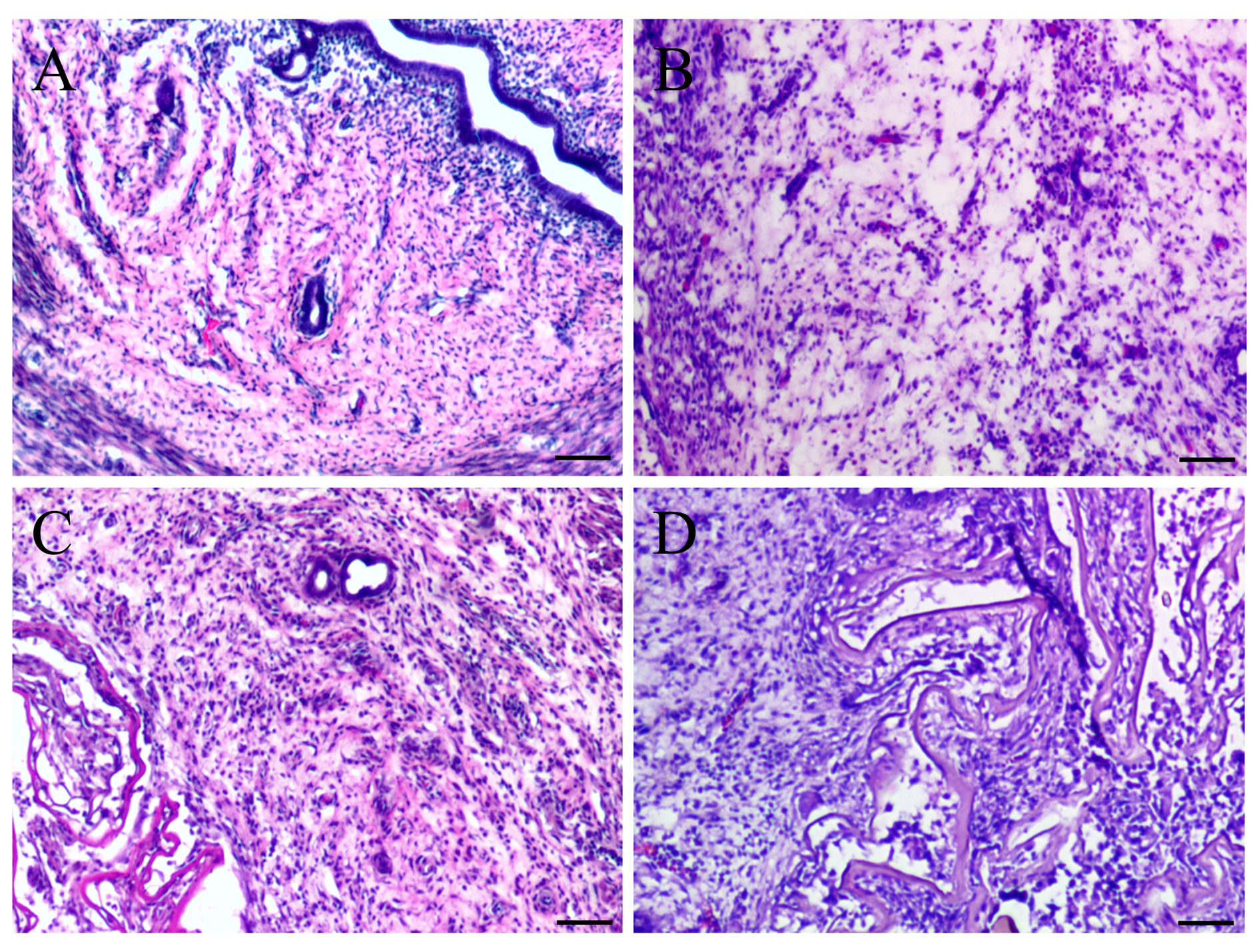

Supplement: Supplementary file 2 — Figure S2. H&E staining of uteri at days 7 post-surgery in control group (A), IUA group (B), DL-AM group (C) and DL-AM+OMECs group (D). Regeneration of endometrium was not found. Bar = 100 μm. (TIF 6333 kb) [file 13287_2019_1179_MOESM2_ESM.tif]

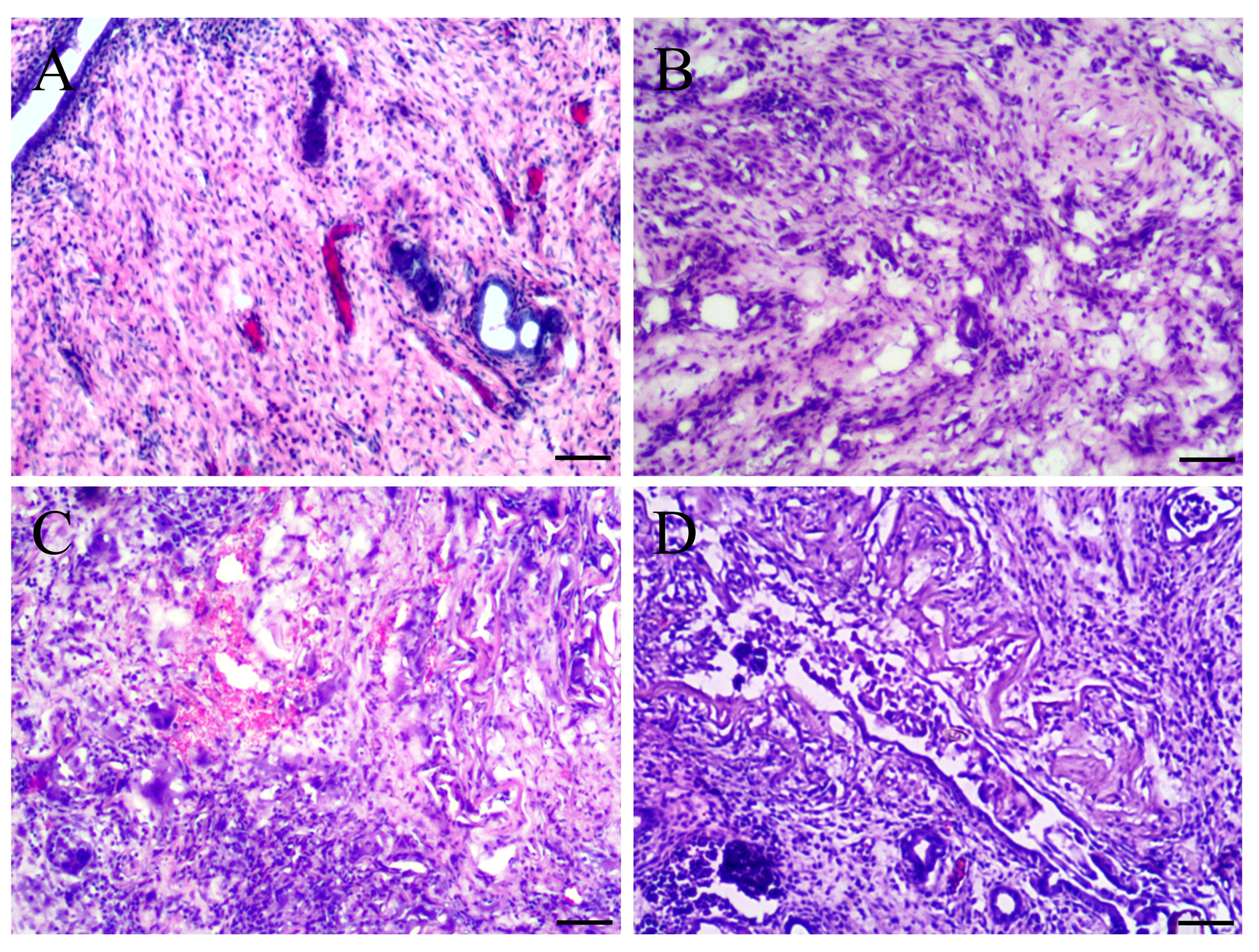

Supplement: Supplementary file 3 — Figure S3. H&E staining of uteri at days 14 post-surgery in control group (A), IUA group (B), DL-AM group (C) and DL-AM+OMECs group (D). Regeneration of endometrium was found in DL-AM+OMECs group. Bar = 100 μm. (TIF 6258 kb) [file 13287_2019_1179_MOESM3_ESM.tif]

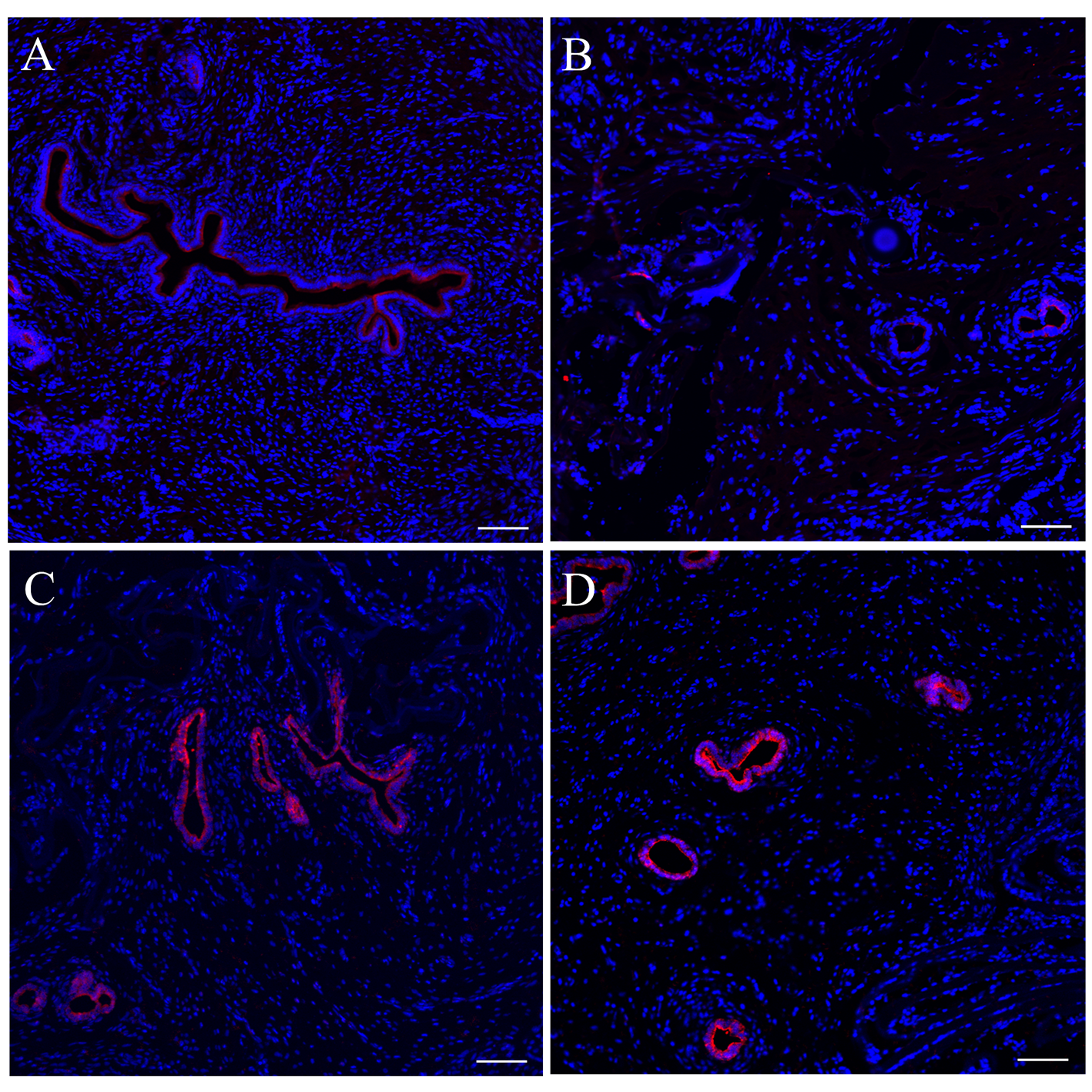

Supplement: Supplementary file 4 — Figure S4. Immunofluorescent staining of CK-18 in control group (A), IUA group (B), DL-AM group (C) and DL-AM+OMECs group (D) after 3 days of operations. Bar = 100 μm. (TIF 2882 kb) [file 13287_2019_1179_MOESM4_ESM.tif]

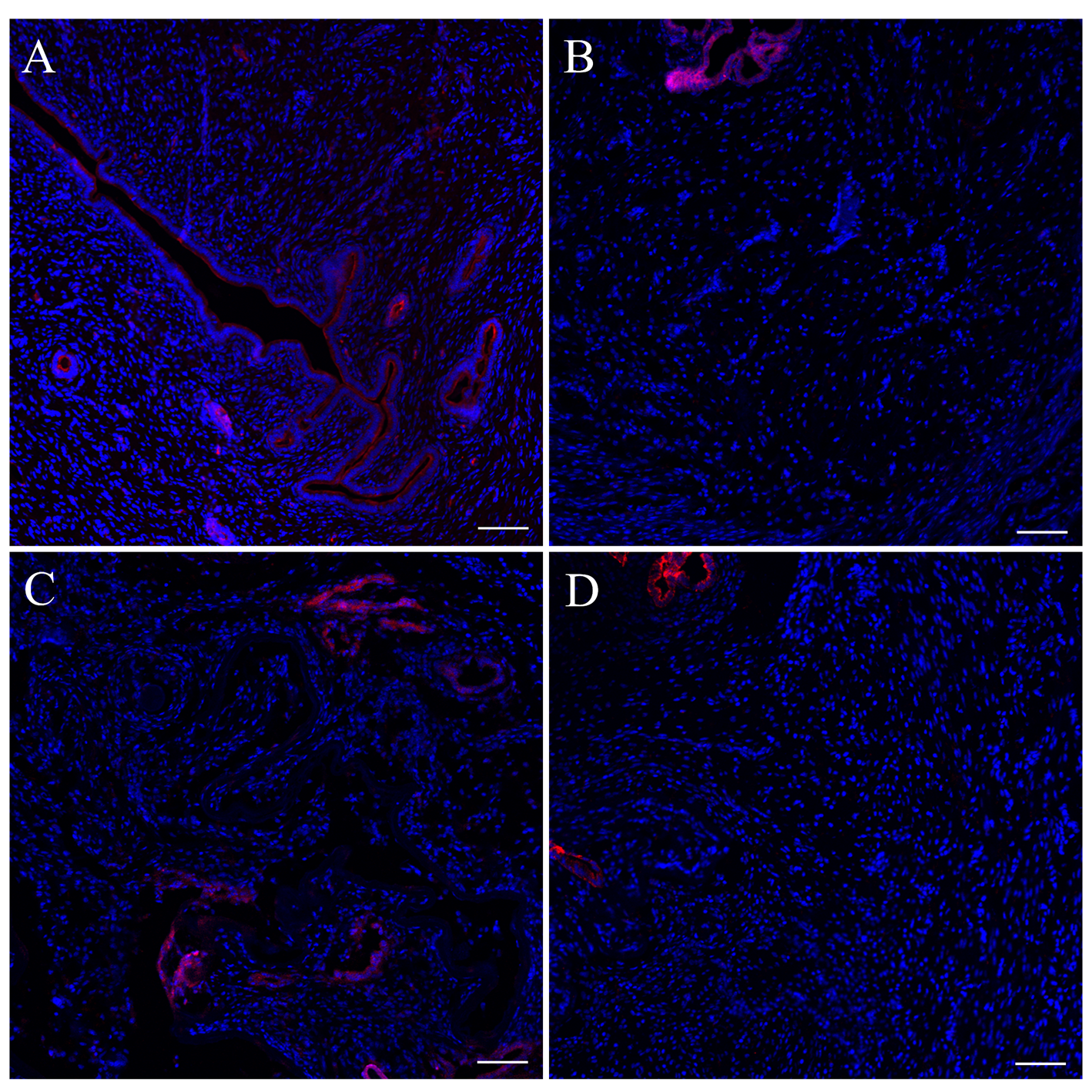

Supplement: Supplementary file 5 — Figure S5. Immunofluorescent staining of CK-18 in control group (A), IUA group (B), DL-AM group (C) and DL-AM+OMECs group (D) after 7 days of operations. Bar = 100 μm. (TIF 2969 kb) [file 13287_2019_1179_MOESM5_ESM.tif]

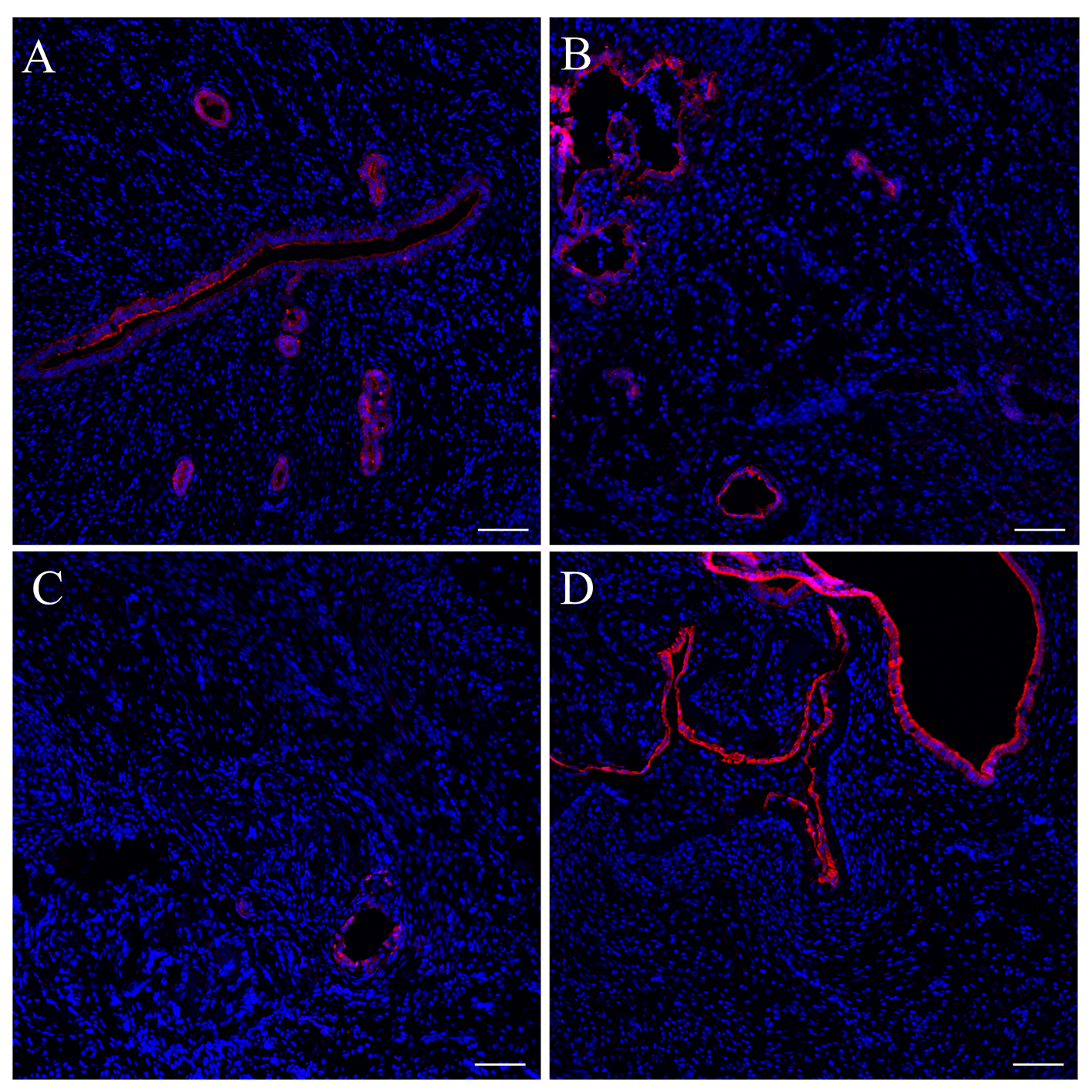

Supplement: Supplementary file 6 — Figure S6. Immunofluorescent staining of CK-18 in control group (A), IUA group (B), DL-AM group (C) and DL-AM+OMECs group (D) after 14 days of operations. Bar = 100 μm. (TIF 2869 kb) [file 13287_2019_1179_MOESM6_ESM.tif]

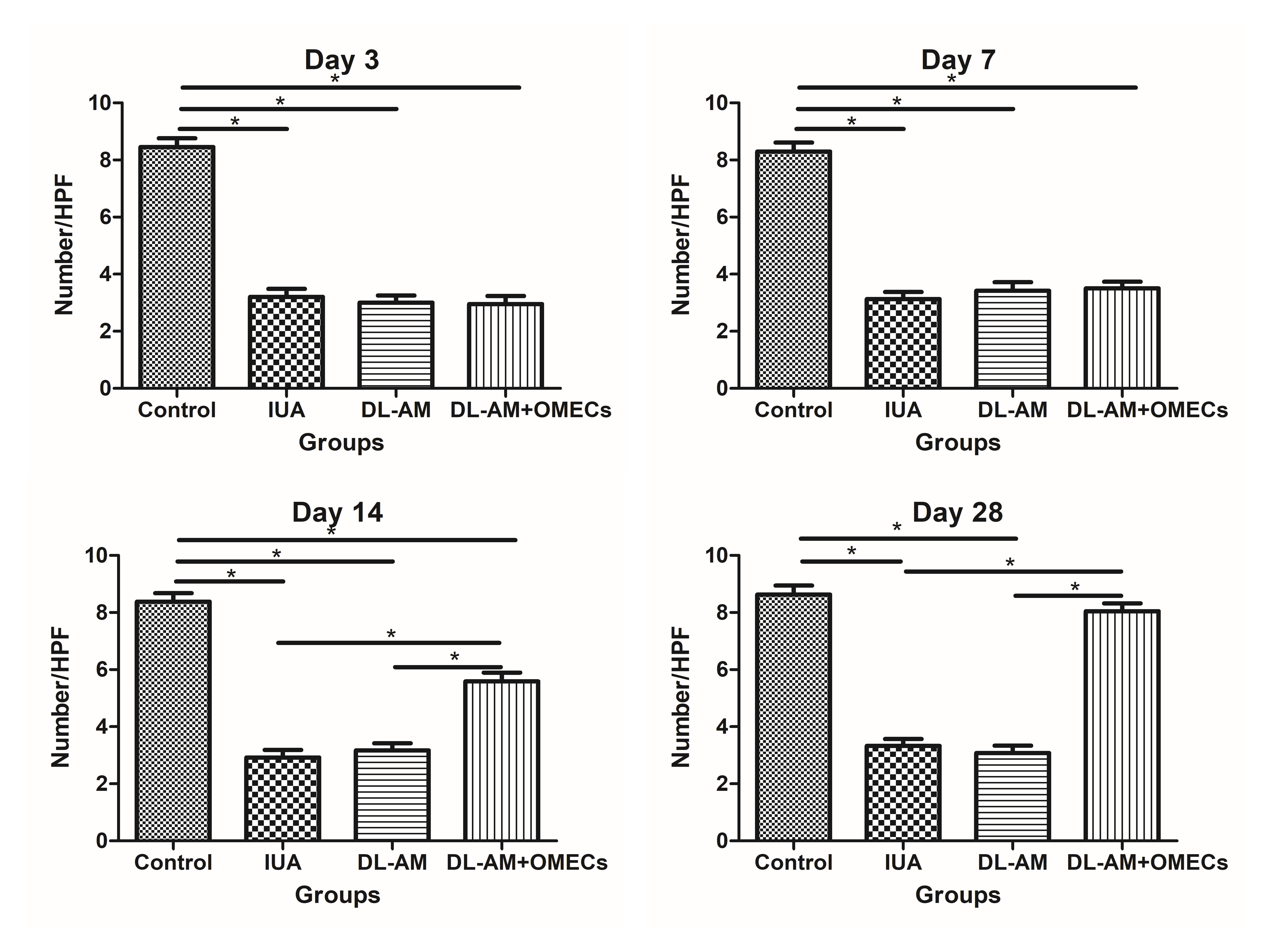

Supplement: Supplementary file 7 — Figure S7. The comparison of the number of endometrial glands in control group, IUA group, DL-AM group and DL-AM+OMECs group after 3, 7, 14 and 28 days of surgeries. *P<0.05. (TIF 4577 kb) [file 13287_2019_1179_MOESM7_ESM.tif]

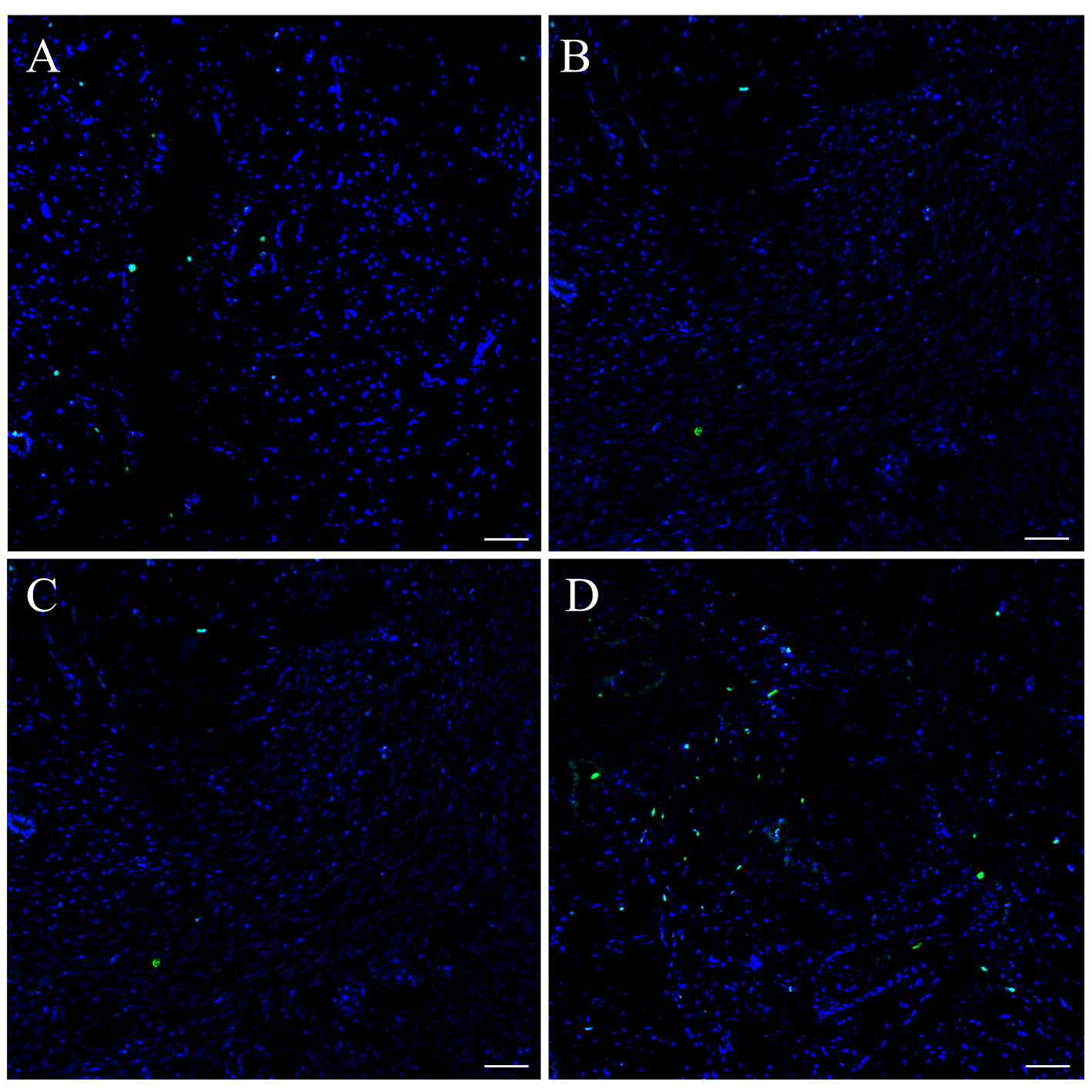

Supplement: Supplementary file 8 — Figure S8. Immunofluorescent staining of Ki-67 after 3 days of operation. Bar = 100 μm. (TIF 1516 kb) [file 13287_2019_1179_MOESM8_ESM.tif]

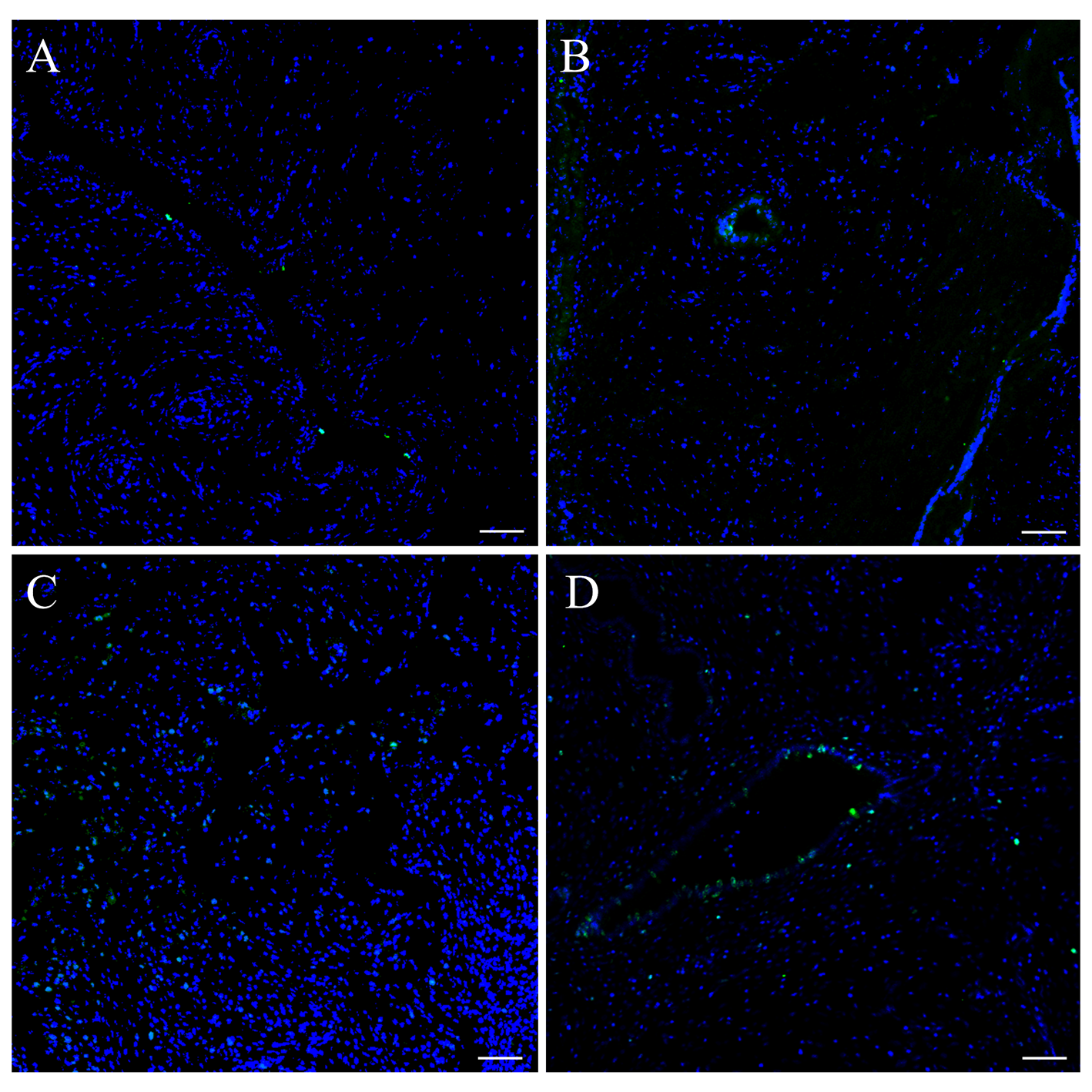

Supplement: Supplementary file 9 — Figure S9. Immunofluorescent staining of Ki-67 after 7 days of operation. Bar = 100 μm. (TIF 1141 kb) [file 13287_2019_1179_MOESM9_ESM.tif]

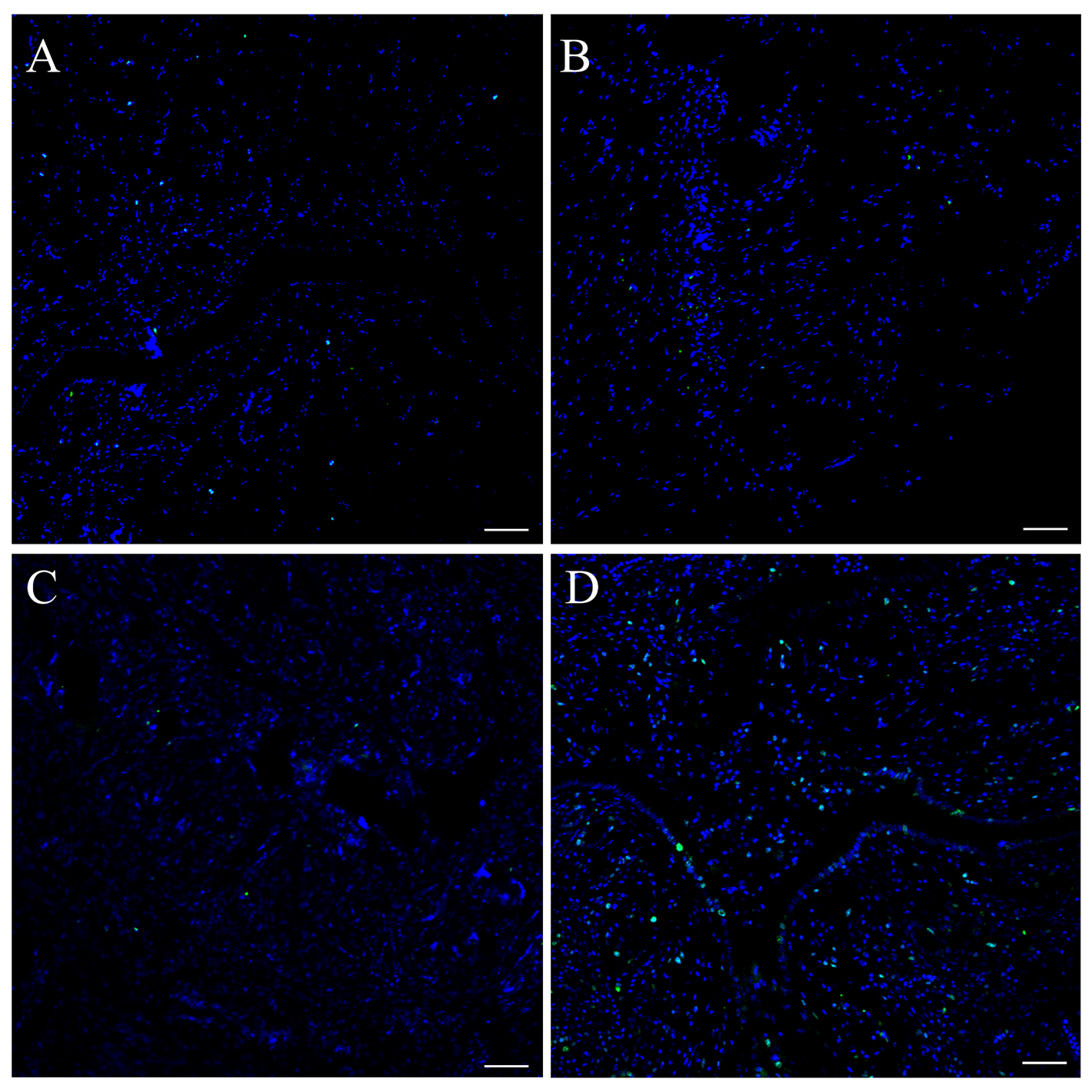

Supplement: Supplementary file 10 — Figure S10. Immunofluorescent staining of Ki-67 after 14 days of operation. Bar = 100 μm. (TIF 1126 kb) [file 13287_2019_1179_MOESM10_ESM.tif]

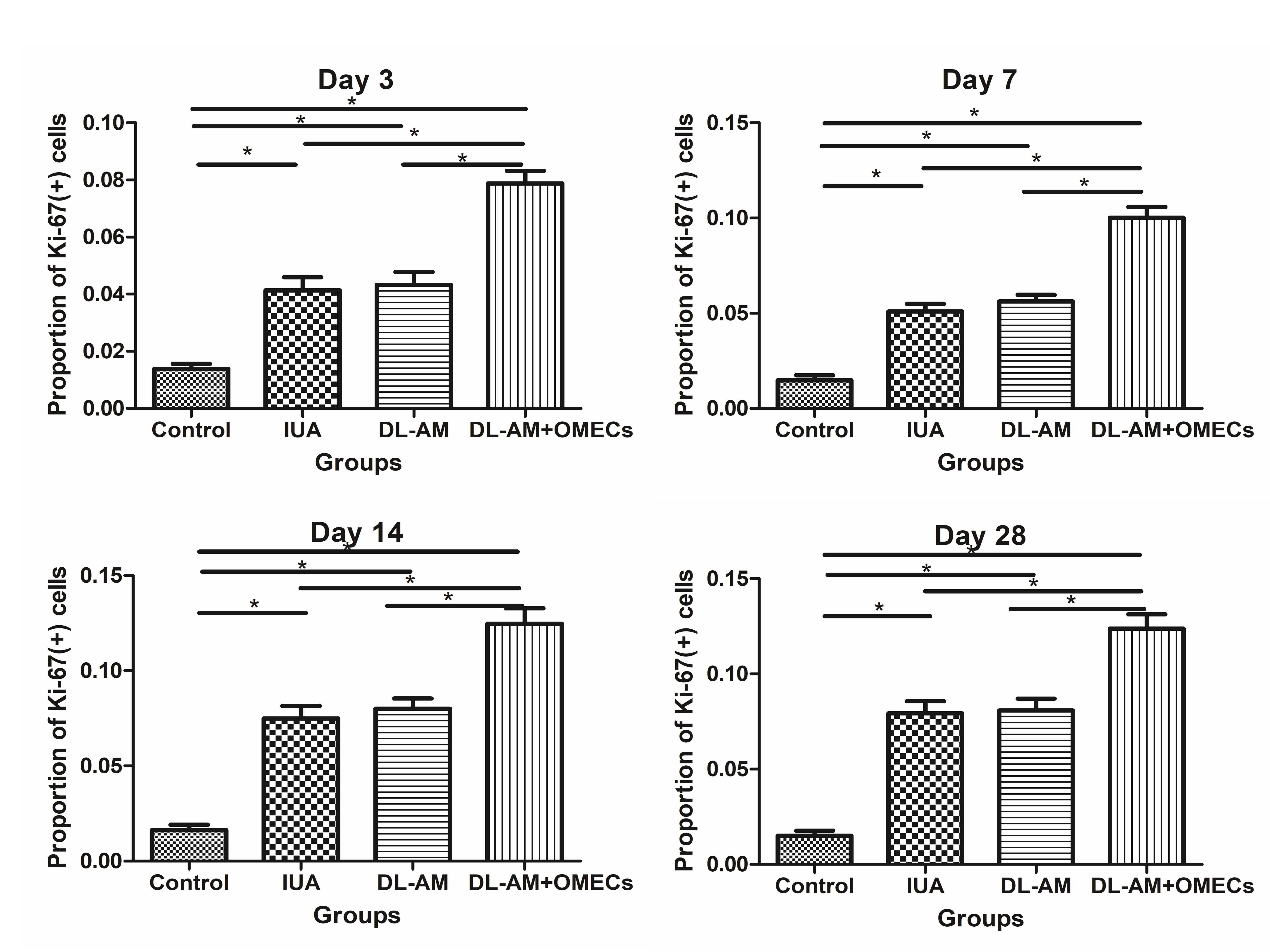

Supplement: Supplementary file 11 — Figure S11. Comparison of the percentage of Ki-67(+) cells in different groups after 3, 7, 14 and 28 days of operation. *P < 0.05. (TIF 4446 kb) [file 13287_2019_1179_MOESM11_ESM.tif]

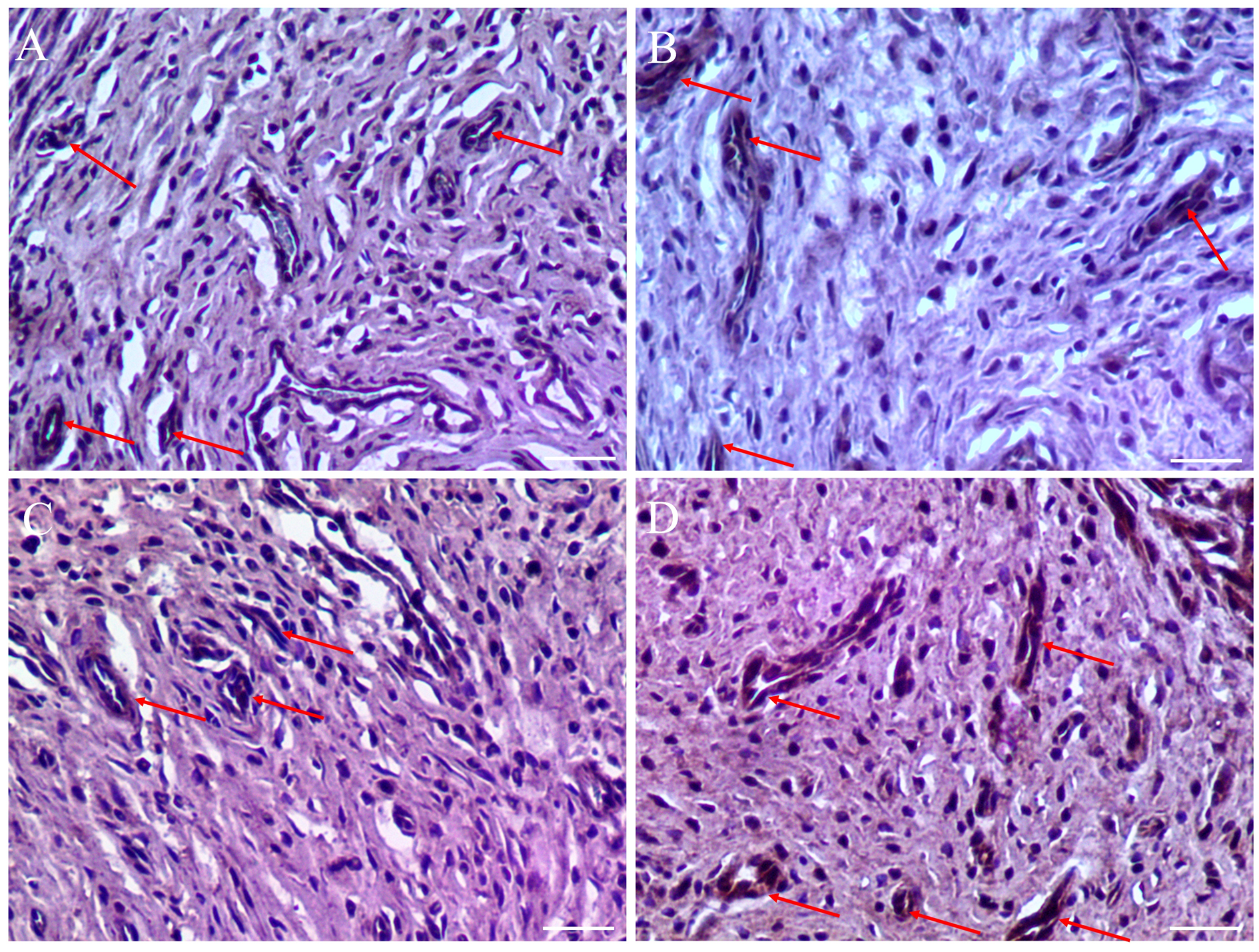

Supplement: Supplementary file 12 — Figure S12. Immunohistochemical staining of CD34 at 3 days after operations. Red arrows indicated microvessels which were positive for CD34. Bar = 100 μm. (TIF 6665 kb) [file 13287_2019_1179_MOESM12_ESM.tif]

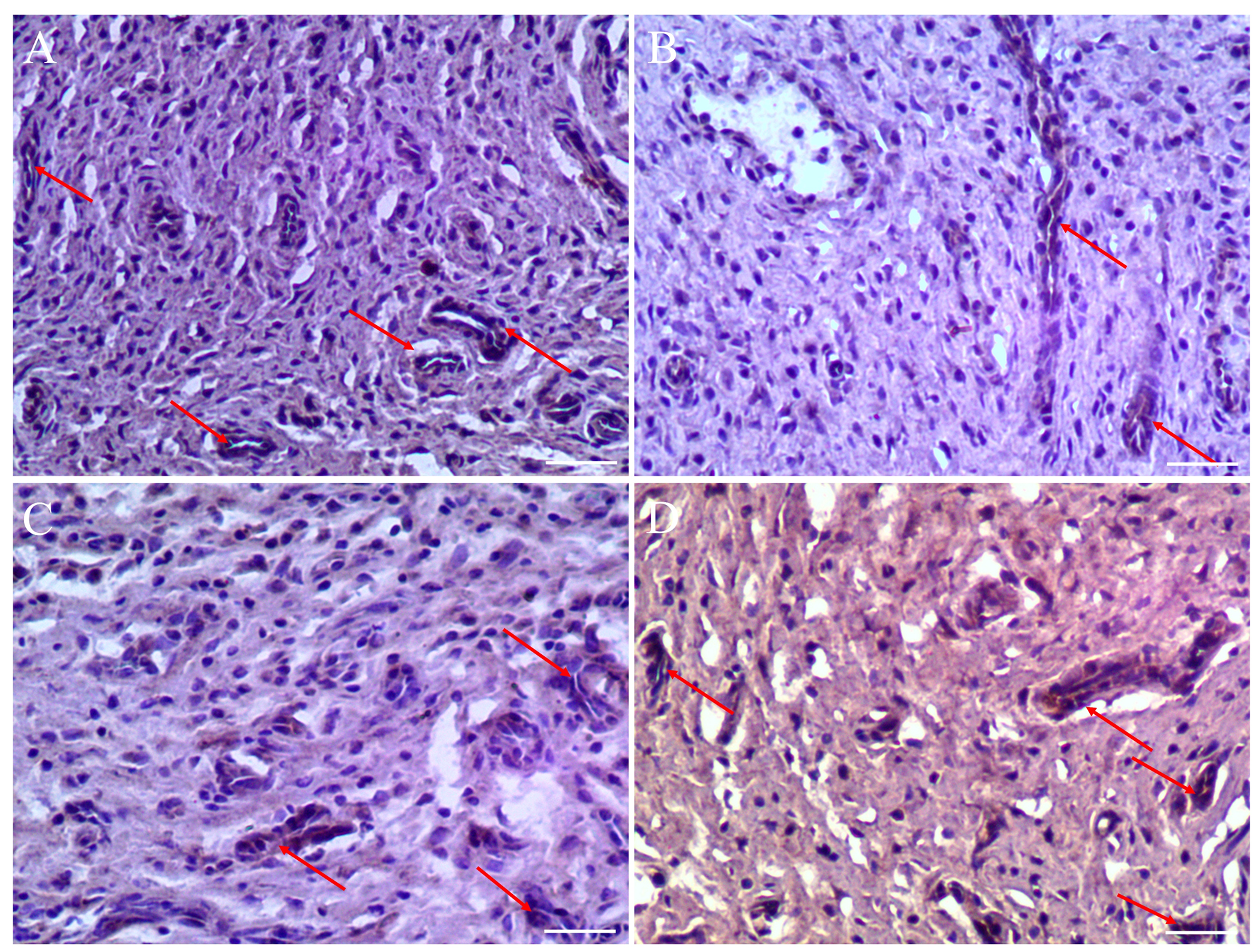

Supplement: Supplementary file 13 — Figure S13. Immunohistochemical staining of CD34 at 7 days after operations. Red arrows indicated microvessels which were positive for CD34. Bar = 100 μm. (TIF 6370 kb) [file 13287_2019_1179_MOESM13_ESM.tif]

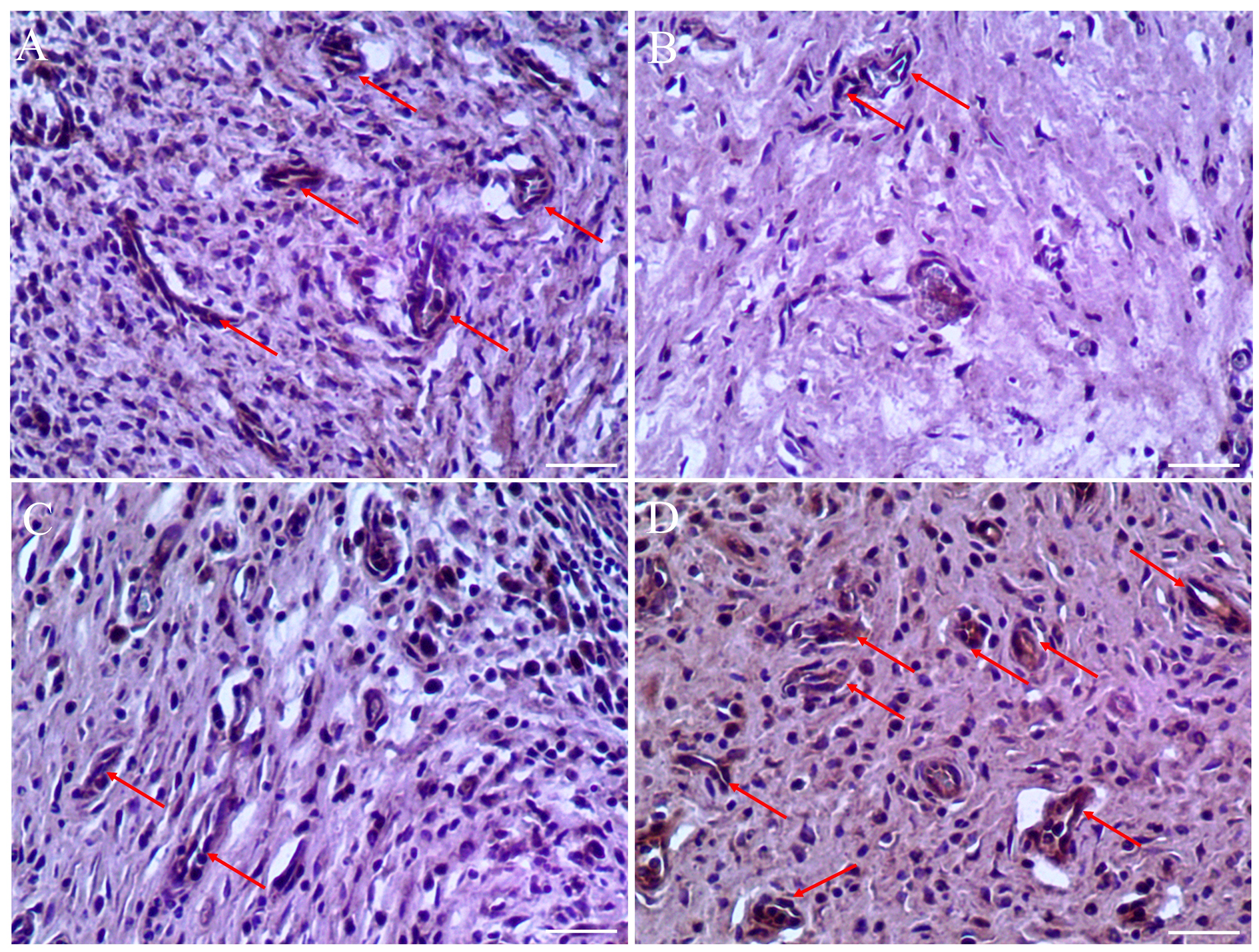

Supplement: Supplementary file 14 — Figure S14. Immunohistochemical staining of CD34 at 14 days after operations. Red arrows indicated microvessels which were positive for CD34. Bar = 100 μm. (TIF 6599 kb) [file 13287_2019_1179_MOESM14_ESM.tif]

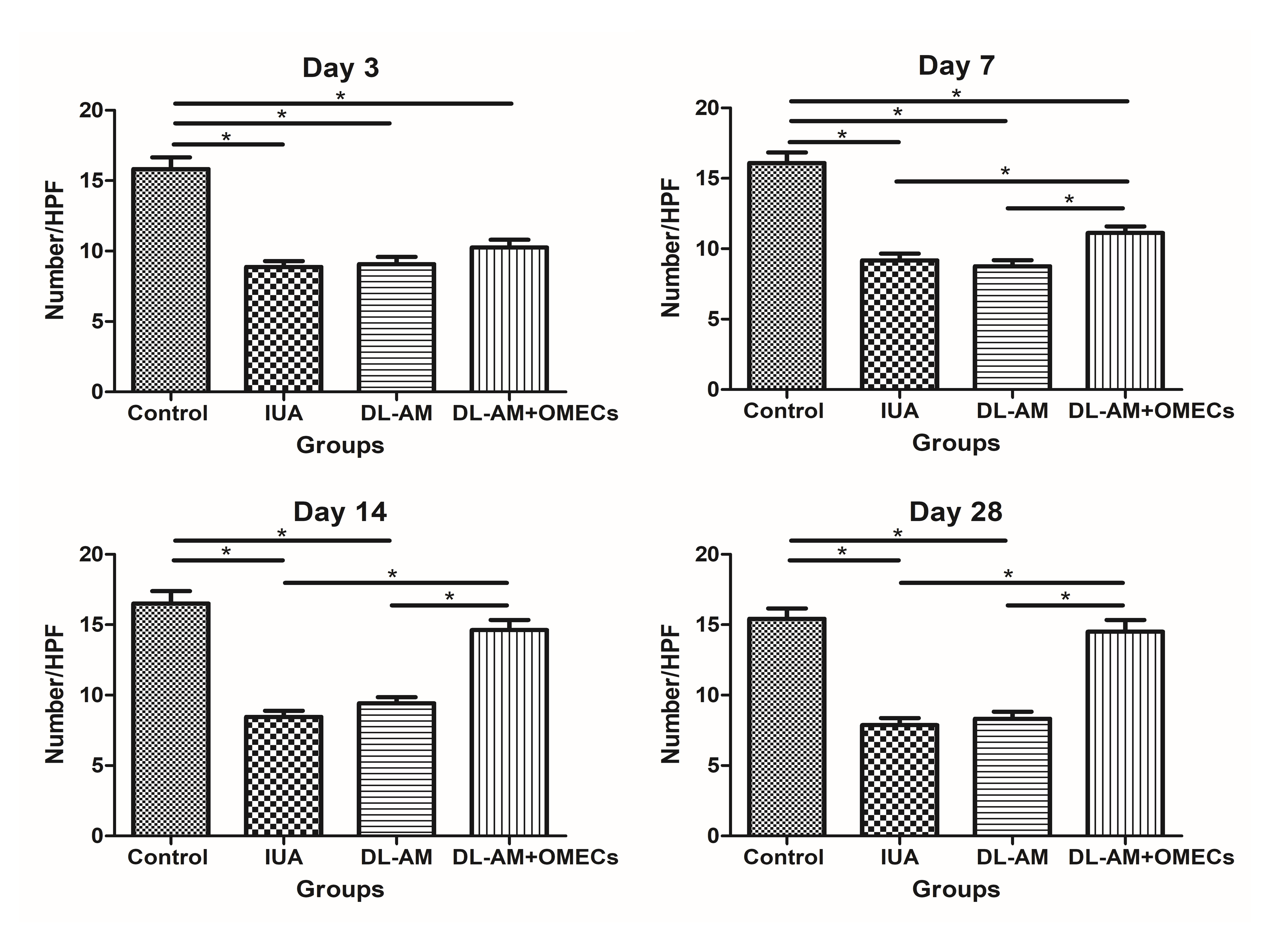

Supplement: Supplementary file 15 — Figure S15. Comparison of the MVD among different groups. *P < 0.05. (TIF 4666 kb) [file 13287_2019_1179_MOESM15_ESM.tif]

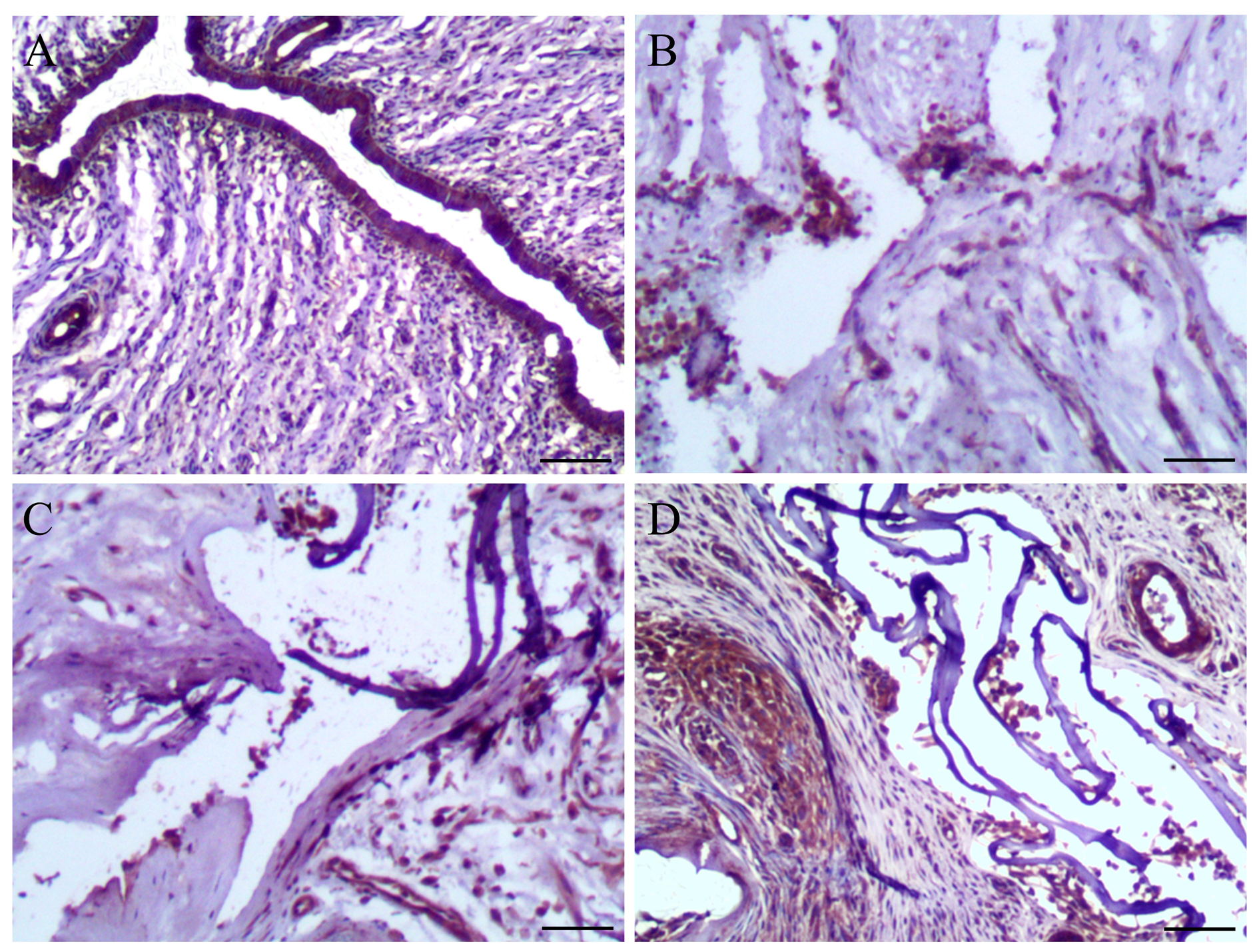

Supplement: Supplementary file 16 — Figure S16. Immunohistochemical staining of VEGF among groups at 3 days after operations. Bar = 100 μm. (TIF 5500 kb) [file 13287_2019_1179_MOESM16_ESM.tif]

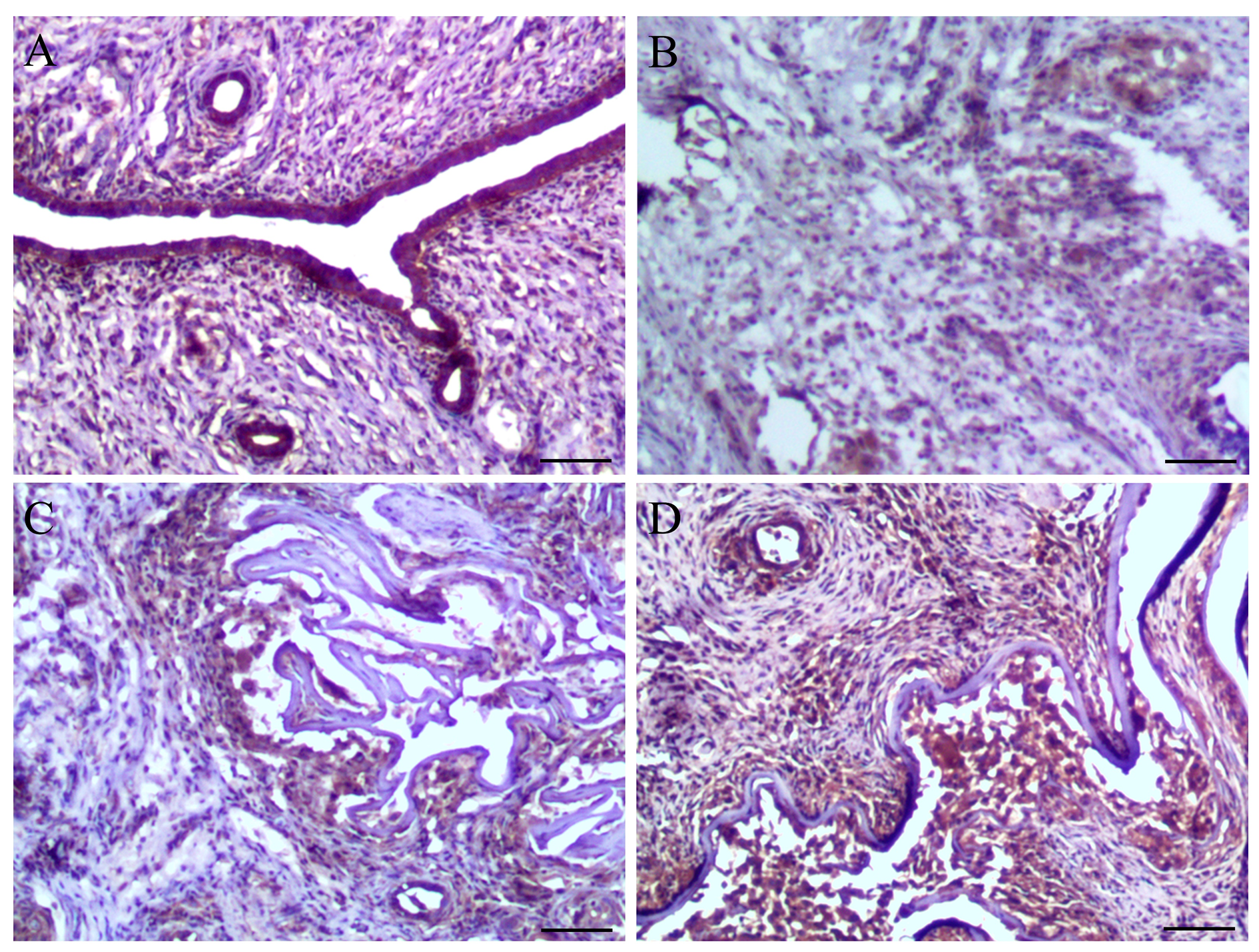

Supplement: Supplementary file 17 — Figure S17. Immunohistochemical staining of VEGF among groups at 7 days after operations. Bar = 100 μm. (TIF 5863 kb) [file 13287_2019_1179_MOESM17_ESM.tif]

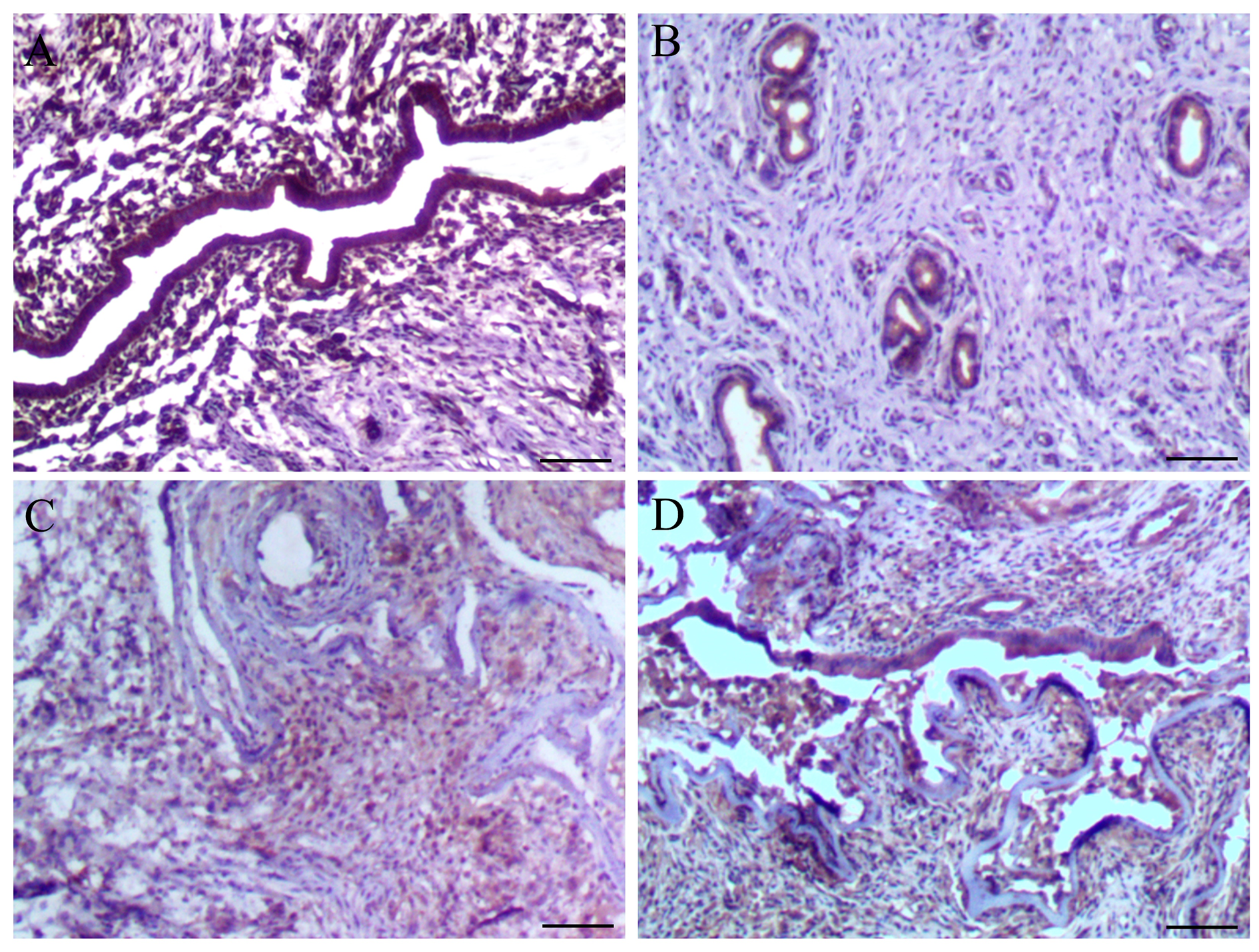

Supplement: Supplementary file 18 — Figure S18. Immunohistochemical staining of VEGF among groups at 14 days after operations. Bar = 100 μm. (TIF 5741 kb) [file 13287_2019_1179_MOESM18_ESM.tif]

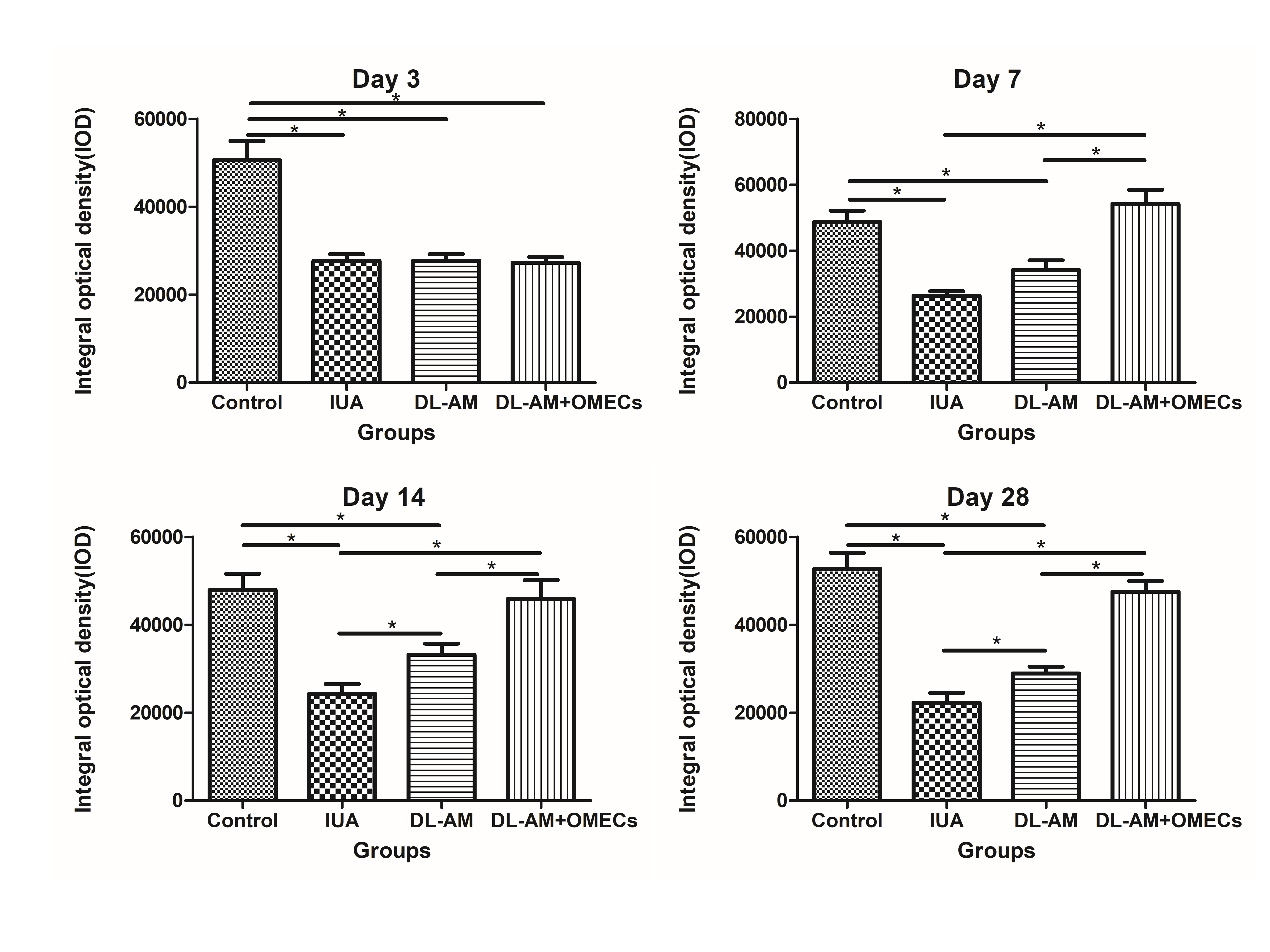

Supplement: Supplementary file 19 — Figure S19. Histogram of comparison of VEGF expression among different groups. Bar = 50 μm.*P < 0.05. (TIF 4586 kb) [file 13287_2019_1179_MOESM19_ESM.tif]
